# Exploring the effects of dietary methionine supplementation on European seabass mucosal immune responses against *Tenacibaculum maritimum*

**DOI:** 10.3389/fimmu.2025.1513516

**Published:** 2025-01-22

**Authors:** Inês Carvalho, Diogo Peixoto, Inês Ferreira, Diego Robledo, Lourenço Ramos-Pinto, Rodolfo Miguel Silva, José Fernando Gonçalves, Marina Machado, Carolina Tafalla, Benjamin Costas

**Affiliations:** ^1^ Centro Interdisciplinar de Investigação Marinha e Ambiental, Universidade do Porto, Matosinhos, Portugal; ^2^ Instituto de Ciências Biomédicas Abel Salazar, Universidade do Porto, Porto, Portugal; ^3^ The Roslin Institute and Royal (Dick) School of Veterinary Studies, The University of Edinburgh, Edinburgh, United Kingdom; ^4^ Department of Genetics, Universidade de Santiago de Compostela, Santiago de Compostela, Spain; ^5^ Centro de Investigación en Sanidad Animal, Instituto Nacional de Investigación y Technología Agraria y Alimentaria (CISA-INIA-CSIC), Madrid, Spain

**Keywords:** amino acids, skin, bacterial challenge, RNA sequencing, immunomodulation

## Abstract

**Introduction:**

Dietary methionine supplementation has been shown to enhance immunity and disease resistance in fish. However, excessive intake may lead to adverse effects. The present study aimed to evaluate the immune status of European seabass (*Dicentrarchus labrax*) fed increasing levels of dietary methionine supplementation and to investigate the early immune response to *Tenacibaculum maritimum*.

**Methods:**

For this purpose, juvenile European seabass were fed one of three experimental diets containing methionine at 8.6 mg/g (CTRL), 18.5 mg/g (MET2), and 29.2 mg/g (MET3) for four weeks, followed by a bath challenge with *T. maritimum*.

**Results:**

While higher methionine intake reduced hemoglobin levels, no other significant changes in the immune status were observed. However, after infection, fish fed higher methionine levels exhibited a dose-dependent decrease in the mRNA expression of some proinflammatory genes. Similarly, RNA sequencing analysis of skin tissue revealed an attenuated immune response in the MET2 group at 24 hours post-infection, with few proinflammatory genes upregulated, which intensified at 48 h, potentially due to advanced tissue colonization by *T. maritimum*. The MET3 group displayed the least pronounced immune response, along with the enrichment of some immune-related pathways among the downregulated transcripts. These findings, together with the lower mRNA expression of proinflammatory genes in the head kidney and the higher mortality rates observed in this group, suggest a potential impairment of the immune response.`

**Discussion:**

Overall, these findings indicate that dietary methionine supplementation may significantly influence both systemic and local immune responses in European seabass, highlighting the need for careful consideration when supplementing diets with methionine.

## 1 Introduction

Metabolic, physiological and neuronal homeostasis, as well as disease resistance, greatly depend on essential nutrients found in food, including amino acids, fatty acids, vitamins and minerals (1). This close interplay between nutrition and health, emphasizes the importance of ensuring a well-balanced diet in livestock farming (2). In modern aquaculture, beyond providing essential nutritional requirements, the supplementation of diets with functional ingredients, such as prebiotics, probiotics, nucleotides, pigments, vitamins and amino acids, has become a widespread strategy to improve fish robustness (3), ultimately reducing their susceptibility to diseases (4, 5).

In this context, methionine, an essential sulfur-containing amino acid, plays a pivotal role in supporting various physiological functions (6). As a part of the one-carbon cycle, methionine is converted to S-adenosylmethionine (SAM), which is the universal cellular donor of the methyl (-CH3) group, and later to S-adenosylhomocysteine (SAH) (7). SAM participates in various processes, including the methylation of proteins, DNA, RNA, histones and phospholipids (8). Therefore, methionine is considered the major contributor to epigenetic regulation among all amino acids (9). By participating in DNA methylation, it regulates gene expression and interferes with cellular functions, including proliferation, differentiation, maintenance and apoptosis (10). A high abundance of SAM is indicative of favorable conditions for methylation processes, whereas a high abundance of the methylation byproduct, SAH, suggests a limited cellular capacity to support methylation reactions. Therefore, the SAM: SAH ratio has been proposed as a “methylation index” and disturbances in it have been associated with health conditions and diseases (11). Methionine also participates in the biosynthesis of polyamines through the aminopropylation pathway, such as spermidine and spermine, which are essential for cell proliferation (12). Additionally, within the transsulfuration pathway, methionine is a precursor of cysteine, one of the three amino acids that compose glutathione (GSH), a free radical scavenger, thereby protecting cells from oxidative stress (13).

The immunomodulatory potential of dietary methionine supplementation has been extensively reviewed in mammalian and poultry production (14, 15) and several studies in fish have recently addressed this issue (16–22). Through the same pathways described in mammals, *in vitro* methionine supplementation has been found to improve the fish’s cell viability, polyamine production and methionine-related gene expression upon inflammatory stimulus (21). *In vivo* dietary supplementation (up to 2x the requirement level) similarly pointed to an overall improved immune status and enhanced immune response to bacterial infection of European seabass (*Dicentrarchus labrax*) (17–19). However, the impact of methionine intake on mucosal tissues, particularly the skin, remains poorly explored (23, 24). Given that mucosal tissues act as the primary barrier against invading pathogens (25), investigating their defense mechanisms is of particular importance.

Despite the potential benefits, excessive dietary methionine intake should be considered since it has been associated with adverse effects (26–29), including hyperhomocysteinemia, reduced body weight, increased cholesterol levels (30) and impaired liver function (31). Thus, providing adequate dietary methionine supplementation is essential to support the fish’s normal physiological condition (30).

Hence, the current study aimed firstly to assess the immune status of European seabass (*Dicentrarchus labrax*) fed increasing dietary methionine supplementation levels and subsequently the inflammatory response against *Tenacibaculum maritimum*, examining both the systemic and local immune responses. The selection of this Gram-negative bacterium was based on its infection routes. This bacterium primarily targets mucosal surfaces, specifically the skin tissue and mucus, leading to the ulcerative disease tenacibaculosis (32, 33). Exploring differences in the skin immune response of fish fed high levels of methionine is important for expanding our knowledge on how methionine intake modulates genetic resistance against pathogens.

## 2 Materials and methods

### 2.1 Experimental diets

Three isonitrogenous (45% crude protein) and isolipidic (16% crude fat) diets, differing in methionine content, were formulated and manufactured by Sparos Lda. (Olhão, Portugal) (**Table 1**). The control (CTRL) diet was specifically formulated to meet the amino acid requirements of European seabass (34) and two experimental diets, identical to the CTRL, were supplemented with DL-methionine at 2 or 3% of feed weight (MET2 and MET3, respectively) at the expenses of wheat gluten and wheat meal. The resulting methionine concentrations were 8.6, 18.5 and 29.2 mg g^-1^ dry matter, respectively (**Table 2**), meaning an increase of approximately 110% and 220% in the MET2 and MET3 diets, respectively, compared to the CTRL diet.

**Table 1:** Ingredients and chemical composition of the experimental diets.

Ingredients	CTRL	MET2	MET3
	%		
Fishmeal LT70^1^	11.0	11.0	11.0
Fishmeal 60^2^	17.0	17.0	17.0
Soy protein concentrate^3^	12.0	12.0	12.0
Wheat gluten^4^	7.0	6.4	5.8
Corn gluten meal^5^	4.0	4.0	4.0
Soybean meal 44^6^	15.0	15.0	15.0
Rapeseed meal^7^	6.0	6.0	6.0
Wheat meal^8^	10.0	9.6	9.2
Fish oil^9^	8.5	8.5	8.5
Rapeseed oil^10^	5.0	5.0	5.0
Vitamin and mineral premix^11^	1.0	1.0	1.0
Brewer”s yeast^12^	3.0	3.0	3.0
Soy lecithin^13^	0.5	0.5	0.5
DL-Methionine^14^	0.0	1.0	2.0
Total	100	100	100
Proximate Analysis (% dry weight)			
Ash (g/100g)	8.5	8.5	8.6
Energy (kJ/g)	22.5	22.7	22.7
Fat (g/100g)	17.4	17.7	17.7
Protein (g/100g)	49.5	49.6	50.0

^1^LT70 Steam Dried, 70.7% crude protein (CP), 8.1% crude fat (CF), Pesquera Diamante, Peru.

^2^COFACO 60: 62.3% CP, 8.4% CF, COFACO, Portugal.

^3^Soycomil P: 63% CP, 0.8% CF, ADM, The Netherlands.

^4^VITAL: 83.7% CP, 1.6% CF, ROQUETTE Frères, France.

^5^Corn gluten meal: 61% CP, 6% CF, COPAM, Portugal.

^6^Dehulled solvent extracted soybean meal: 47% CP, 2.6% CF, CARGILL, Spain.

^7^Defatted rapeseed meal: 34% CP, 2% CF, Premix Lda, Portugal.

^8^Wheat meal: 10.2% CP; 1.2% CF, Casa Lanchinha, Portugal.

^9^SAVINOR UTS, Portugal.

^10^Henry Lamotte Oils GmbH, Germany.

^11^20 PREMIX Lda, Portugal: Vitamins (IU or mg/kg diet): DL-alpha tocopherol acetate, 100 mg; sodium menadione bisulfate, 25mg; retinyl acetate, 20000 IU; DL-cholecalciferol, 2000 IU; thiamin, 30mg; riboflavin, 30mg; pyridoxine, 20mg; cyanocobalamin, 0.1mg; nicotinic acid, 200mg; folic acid, 15mg; ascorbic acid, 500mg; inositol, 500mg; biotin, 3mg; calcium pantothenate, 100mg; choline chloride, 1000mg, betaine, 500mg. Minerals (g or mg/kg diet): copper sulfate, 9mg; ferric sulfate, 6mg; potassium iodide, 0.5mg; manganese oxide, 9.6mg; sodium selenite, 0.01mg; zinc sulfate,7.5mg; sodium chloride, 400mg; excipient wheat middlings.

^12^PREMIX Lda, Portugal.

^13^Lecico P700IPM, LECICO GmbH, Germany.

^14^DL-Methionine for Aquaculture: 99% Methionine, Evonik Nutrition & Care GmbH, Germany.

**Table 2:** Amino acid composition (mg g^-1^ DW) of the experimental diets.

Amino acids	CTRL	MET2	MET3
	mg AA g^-1^ DW diet		
Alanine	25.1	25.7	25.4
Arginine	26.4	27.4	26.9
Aspartic Acid	38.5	40.8	38.6
Glutamic Acid	86.4	89.2	85.4
Glycine	28.9	29.5	29.1
Histidine	10.4	10.6	10.7
Isoleucine	18.1	17.9	17.8
Leucine	35.0	35.6	34.8
Lysine	25.0	24.6	25.1
Phenylalanine	21.0	20.6	20.6
Proline	31.3	31.2	31.3
Serine	21.1	21.8	20.9
Threonine	17.2	17.9	17.0
Tyrosine	14.0	14.5	14.3
Valine	20.4	20.7	21.0
Cysteine + Cystine	5.6	5.7	5.6
**Methionine**	**8.63**	**18.5**	**29.2**

Methionine final concentration (mg methionine g_-1_ DW diet) in the three experimental diets is highlighted in bold.

Main ingredients were ground (below 250 μm) in a micropulverizer hammer mill (SH1; Hosokawa Micron, B.V., Doetinchem, The Netherlands). Powder ingredients and oils were then mixed according to the target formulation in a paddle mixer (RM90; Mainca, S.L., Granollers, Spain). All diets were manufactured by temperature-controlled extrusion (pellet sizes: 1.5 mm) by means of a low-shear extruder (P55; Italplast, S.r.L., Parma, Italy). Upon extrusion, all feed batches were dried in a convection oven (OP 750-UF; LTE Scientifics, Oldham, UK) for 4 h at 45°C. Proximate composition analyses were conducted by the following methods: dry matter, by drying at 105°C for 24 h; ash, by combustion at 550°C for 12 h; crude protein (N × 6.25), by a flash combustion technique followed by gas chromatographic separation and thermal conductivity detection (LECO FP428); fat, after petroleum ether extraction, by the Soxhlet method; gross energy, in an adiabatic bomb calorimeter (IKA). Formulation and proximate composition of experimental diets are presented in **Table 1**.

Diets were also analyzed for total amino acids content by a certified laboratory (Eurofins Food & Agro, Portugal) and according to the method ISO 13903:2005; EU 152/2009. The total amino acids profile of the experimental diets is presented in **Table 2**.

### 2.2 Experimental design

The trial was conducted at the Institute of Biomedical Sciences Abel Salazar, ICBAS (University of Porto, Portugal), where European seabass juveniles (5.20 ± 0.69 g) from a certified commercial fish farm (AVRAMAR, Spain) were reared by trained scientists (following FELASA category C recommendations). The experiment complied with the national rules and followed the European Union Directive 2010/63/EU on the protection of animals used for scientific purposes. Upon arrival at ICBAS, fish were randomly distributed into nine tanks of 110 litters (50 fish per tank) of a recirculation system and acclimated under the following rearing conditions: temperature 22 ± 1°C, salinity 30 ± 1 and photoperiod 13/11-hours light/dark cycle. During the acclimatization period to the rearing facilities, fish were fed twice per day at an average ratio of 3% biomass with the CTRL diet. Thereafter, the trial was initiated by the introduction of the two dietary treatments (MET2 and MET3) in triplicate tanks each while the remaining three tanks continued to be fed the CTRL diet. Fish were fed the experimental diets during four weeks at an average ratio of 3% biomass.

After feeding on the experimental diets for four weeks, a group of fish were sampled while the remaining were either bath infected with *T. maritimum* (1 x 10^6^ colony forming units (CFU) ml^-1^) or sham infected to serve as negative control. After the bath infection procedure, fish from the infected groups were split into two groups: one to perform a time-course trial to study host immune responses and the other to monitor the mortality over one week. The infected fish intended for the time-course trial (30 per group, 10 per replicate) were placed back in the tanks of the system used for the feeding trial and sampled at 4, 24 and 48 h after. The mortality assessment took place in similar RAS system composed by 12 independent tanks: 6 of them were used for the sham-infected fish (10 fish per tank, 2 tanks per treatment), and the other 6 tanks were used for the infected fish (20 fish per tank, 2 tanks per treatment). Fish were fed according to the previous regimen and the mortality was recorded daily. After euthanasia of the moribund fish, the presence of *T. maritimum* in the head kidney was checked by growing the inoculum from the collected samples onto marine agar plates.

### 2.3 Bacterial bath challenge

For the bacterial challenge, *T. maritimum* (strain ACC13.1) isolated from *Senegalese sole* in a local fish farm (Portugal) (35) was cultured at 22°C in marine agar for 48 h, followed by inoculation in marine broth overnight at the same temperature with continuous strong shaking. To prepare the inoculum for the bath challenge, exponentially growing bacteria were collected by centrifugation (4000 × *g* for 10 min at 4°C), re-suspended in sterile marine broth to a concentration of 2.24 × 10^9^ CFU ml^-1^ (optical density (OD) read at 600 nm in a cuvette spectrophotometer). The bacterial concentration was adjusted with the predetermined growth curve for this specific strain: y = 2 x 10^8^ *x* + 4 x 10^7^ (36). The inoculum was then added to tanks with a water volume of 10 liters to a final concentration of 1 × 10^6^ CFU ml^-1^ (10 fish per tank). This dosage was selected according to the outcomes of pre-challenges previously performed on the same batch of fish to find the median lethal dose, LD_50_. The immersion challenge was stopped after 2 h by moving the fish from the infection tanks to the clean systems. At this point and until the end of the trial, the water temperature was increased to 24.0 ± 0.5°C to mimic the rise of temperature that triggers piscine outbreaks in aquaculture systems.

### 2.4 Samples collection

There were four sampling points, one before the bacterial bath challenge and then at 4, 24 and 48 h post-infection (hpi). At each point, three fish per tank (9 per treatment) were sampled. They were euthanized via anesthetic overdose, 0.7 ml/l (2-phenoxyethanol; Merck, ref. 807291, Germany) and skin mucus samples were gently collected by scraping the dorsolateral surface of the fish. The collected skin mucus samples were frozen in liquid nitrogen and stored at -80°C until further assessment of innate immune parameters. Immediately after skin mucus collection, fish weight was recorded and blood samples from the caudal vessels were taken. Blood smears were prepared from homogenized blood and air-dried for differential leucocyte counts. Single organ samples of skin tissue, head kidney and intestine were also taken. Both skin tissue and head kidney were submerged in RNA later (with a proportion of 1/10 w/v), kept at 4°C for the first 24 h and then stored at -20°C until processing for RNA sequencing and quantitative PCR, respectively. The intestine was flash-frozen in liquid nitrogen and stored at -80°C until antioxidant capacity assessment.

### 2.5 Analytical procedures

#### 2.5.1 Hematological parameters

Hematological profile was conducted according to Machado, Azeredo (17). Total white and red blood cell concentrations (WBC and RBC, respectively) were determined in a Neubauer chamber. Hematocrit (Ht) and hemoglobin (Hb) were measured (SPINREACT kit, ref. 1001230, Spain). Thereafter, the mean corpuscular volume (MCV), mean corpuscular hemoglobin (MCH) and mean corpuscular hemoglobin concentration (MCHC) were calculated as previously described (17).

#### 2.5.2 Differential peripheral leucocyte counts

In order to count and categorize leucocytes as lymphocytes, monocytes, neutrophils and thrombocytes, blood smears were fixed with formol-ethanol (10% of 37% formaldehyde in absolute ethanol) for 1 min and stained with Wright’s stain (Hemacolor, Merck). The identification of neutrophils was achieved through the detection of neutrophils’ peroxidase activity, according to the technique described by Afonso, Silva (37). Two hundred leucocytes per slide were counted under oil immersion (1000×). The percentage of each leucocyte type was calculated and multiplied by the total number of WBC to determine the number of cells per milliliter.

#### 2.5.3 Skin mucus innate immune parameters

Due to the limited amount of skin mucus, samples from the same tank were pooled (3 fish per pool). Pools were centrifuged (1500 rpm, 10 min, 4°C) and the supernatant was lyophilized. The resultant powder was dissolved in ultra-pure water and the undissolved material was removed by centrifugation (3500 × *g*, 10 min, 4°C). Mucus protein concentration was measured with a colorimetric kit for detection and quantification of total protein (Thermo Scientific™ PeirceTM BCA Protein Assay Kit), according to the manufacturer’s instructions.

Peroxidase activity was measured according to Quade and Roth (38). Briefly, 15 µl of each sample was diluted in 135 µl of HBSS without Ca^+2^ and Mg^+2^ (Cytiva, USA) in 96-well plates. Then, 50 µl of 20 mM 3,3’,5,5’-tetramethylbenzidine hydrochloride (TMB; Sigma, USA) and 50 µl of 5 mM hydrogen peroxide were added. The color-change reaction was stopped after 2 min by adding 50 µl of 2 M sulfuric acid and the OD (optical density) was read at 450 nm in a Synergy HT microplate reader. HBSS, instead of skin mucus, was used as blank. The peroxidase activity (units mg^-1^ protein) was determined by defining one unit of peroxidase as that which produces an absorbance change of 1 OD.

Protease activity was quantified using an azocasein hydrolysis assay. Shortly, 50 µl of mucus sample was incubated with 50 µl of phosphate buffer (115 mM NaH_2_PO_4_, 13.9 mg ml^-1^, pH 7.0) and 125 µl azocasein (20 mg ml^-1^ in 0.5% NaHCO_3_, pH 8.3). After incubation for 24 h at 22°C in polystyrene microtubes, 250 µl of 10% cold trichloroacetic acid (TCA) was added to each mixture, followed by centrifugation (10,000 × g for 5 min). In a microplate, 100 µl of NaOH (40 mg ml^-1^) was added to 100 µl of the supernatant and the absorbance was read at 450 nm (Synergy HT microplate reader). The percentage of protease activity compared to the reference sample (trypsin solution, 5 mg ml^-1^ in NaHCO_3_, pH 8.3) was calculated.

Lysozyme activity was determined following the turbidimetric assay described by Costas, Conceicao (39). In triplicate, 20 μl of skin mucus samples were added to a flat-bottomed 96-well plate and mixed with 140 μl of a solution of *Micrococcus lysodeikticus* (0.25 mg ml^−1^, 0.05 M sodium phosphate buffer, pH 6.2) previously prepared. The reaction was performed at room temperature (RT) and the absorbance (450 nm) was read in a Synergy HT microplate reader over 15 min at 5 min intervals. The amount of lysozyme (µg mg^-1^ protein) was quantified in the samples using a standard curve formula prepared through serial dilutions of lyophilized hen egg-white lysozyme (Sigma) in the above buffer.

An enzyme-linked immunosorbent assay (ELISA) was performed to determine the mucus immunoglobulin M (IgM) levels. In triplicate, 100 μl of diluted mucus (1:10 in 50 mM Na_2_CO_3_, pH 9.6) were placed in flat-bottomed 96-well plates, and the same volume of sodium carbonate (Na_2_CO_3_) instead of mucus was added to some wells as negative controls. After an incubation of 1 h at RT, the plate wells coated with mucus proteins were filled with 300 μl of blocking buffer (5% low-fat milk in T-TBS, Tris Buffered Saline and 0.1% Tween 20, pH 7.6), and the plates incubated for another hour in the same conditions. Plates were then aspirated and rinsed three times with T-TBS and incubated with 100 μl of a commercial mouse anti-European seabass IgM monoclonal antibody (1:50 in blocking buffer; Aquatic Diagnostics Ltd.). After another incubation period of 1 h at RT, plates were rinsed three times with T-TBS and 100 μl of anti-mouse IgG-HRP secondary antibody (1:1000 in blocking buffer; Sigma) was added to each well. Following another incubation time of 1 h at RT and plates being rinsed three times, 100 μl TMB substrate solution for ELISA was added to each well. After 5 min, 100 μl of 2 M sulfuric acid was pipetted to the microplate to stop the reaction. The OD was read at 450 nm (Synergy HT microplate reader).

#### 2.5.4 Intestine antioxidant capacity

Whole intestine samples were homogenized in potassium phosphate buffer (0.1 M, pH 7.4) at a ratio of 1:10 w/v, using a Precellys Evolution homogenizer. One aliquot of 200 µl of homogenate was taken, mixed with 4% BHT (2,6-Di-tert-butyl-4-methylphenol) in methanol and immediately frozen at -80°C for further analysis of lipid peroxidation (LPO) levels. The remaining sample volume was centrifuged (10,000 × *g*, 4°C, 20 min) and the supernatants were stored at -80°C. Total protein concentration in homogenates was measured with the Thermo Scientific™ PeirceTM BCA Protein Assay Kit, as described by the manufacturer.

Catalase (CAT) activity was assessed by measuring the decrease in hydrogen peroxidase concentration according to Claiborne (40), adapted to microplate. Briefly, 10 µl of the diluted sample (0.7 mg ml^-1^ total protein) and 140 µl of potassium phosphate buffer (0.05 M, pH 7.0) were added to a 96-well UV microplate in triplicates. Quickly, 150 µl of a reaction buffer consisting of a hydrogen peroxide solution (30% H_2_O_2_ in potassium phosphate buffer, 0.05 M, pH 7.0) were pipetted to each well and the absorbance immediately read at 240 nm for 2 min (one read every 15 sec. interval) in a Synergy HT microplate reader. The activity of CAT was expressed as units of enzymatic activity per mg of protein. One activity unit (U) was defined as the amount of enzyme needed to transform 1 µmol of substrate per min under the assay conditions.

The concentration of malondialdehyde (MDA), a compound produced during the peroxidation of lipids and which reacts with thiobarbituric acid (TBA) to form a pink compound, was used as a marker of LPO (41). The aliquot previously made and destined for this assay was mixed with 100 µl of 100% TCA. Then, 0.73% thiobarbituric acid solution (in Tris–HCl 60 mM pH 7.4 with DTPA 0.1 mM) was added. After incubation for 1 h at 100°C, the mixture was centrifuged (11,500 rpm, RT, 5 min), 200 µl of the resultant supernatant transferred to a microplate in triplicates and the absorbance was read at 535 nm (Synergy HT microplate reader). Results were expressed as nanomole MDA per gram of wet tissue (nmol g^-1^), calculated from a calibration curve.

#### 2.5.5 Head kidney gene expression analysis

Head kidney was homogenized in TRIzol Reagent (NZYTech, Lisbon, Portugal) using a Precellys Evolution homogenizer. After this step, 150 µl of chloroform were added at 4°C and the samples were vortexed, followed by a centrifugation at 12,000 × *g* for 15 min at 4°C. In a clean tube, the aqueous phase was mixed with 300 µL of 70% ethanol and placed in NZYSpin Binding columns. The total RNA extraction was conducted with the NZY Total RNA Isolation kit (NZYTech, Lisbon, Portugal) according to the manufacturer’s specifications. The RNA integrity was verified through gel electrophoresis and its purity and concentration were measured using the NanoDrop-1000 spectrophotometer (Thermo Scientific, USA). Complementary DNA (cDNA) was synthesized with the NZY First-Stand cDNA Synthesis Kit (NZYTech, Lisbon, Portugal), following the manufacturer’s instructions. Quantitative PCR (qPCR) assays were performed with the CFX384 Touch Real-Time PCR Detection System (Biorad, Hercules, CA, USA) using 4.4 µl of diluted cDNA, 5 µl of iTaq Universal SYBR Green Supermix^®^ (Biorad, Hercules, CA, USA) and 0.3 µl (10 µM) of each specific primer in a final volume of 10 µl in 384 well plates. Primers were designed with NCBI Primer Blast Tool according to the qPCR restrictions (product size, Tm difference between primers, GC content and self-dimer or cross-dimer formation). The efficiency of each primer pair was determined using serial 2-fold dilutions of randomly pooled cDNA by calculating the slope of the regression line of the cycle thresholds (Ct). Melting curve analysis was performed to confirm that no primer dimers were amplified. The standard cycling conditions were 95°C for 10 min, followed by 40 cycles of 95°C for 15 s, 1 min at the specific primer annealing temperature and 95°C extension for 15 s. All reactions were carried out in duplicate. The target gene means were normalized using the delta-delta Ct method. The 40S ribosomal subunit (*40s*) and the elongation factor 1α (*ef1a*) were used as housekeeping genes in the normalization procedure.

Accession number, efficiency values, annealing temperature, product length and primer sequences are displayed in the **Supplementary File S1**, **Supplementary Table S1**.

### 2.6 Skin tissue RNA extraction and sequencing

Samples were selected according to available data on skin transcriptomic responses against *T. maritimum* (42). Therefore, total RNA extraction was performed in skin tissue samples collected before the infection and at 24 and 48 hpi with NZY Total RNA Isolation kit (NZYTech, Lisbon, Portugal) according to the manufacturer’s instructions after tissue homogenization in TRIzol Reagent (Invitrogen). The RNA integrity was verified through gel electrophoresis and its purity and concentration were measured with the Invitrogen Qubit 3.0 Fluorometer using the Qubit RNA HS Assay Kit (ThermoFisher Scientific). Pools of 3 samples per tank were made and the RNA concentration and quality of each pool were measured as described above. Samples were barcoded and sent to Novogene (Cambridge, UK) for sequencing. At their arrival, samples were subjected to an RNA quality control protocol, which all passed with RNA integrity numbers (RIN) greater than 9.3 (average RIN of 9.94). A total of 27 cDNA libraries were built, considering three dietary treatments (CTRL, MET2 and MET3), three sampling times (0, 24 and 48 hpi) and three biological replicates per condition (each replicate a pool of three fish from the same tank). Libraries were built using Illumina’s TruSeq stranded mRNA kit and sequenced on an Illumina Novaseq 6000 instrument as PE150 reads.

#### 2.6.1 Differential expression analysis

The sequencing output quality was assessed using FastQC v0.12.0 (https://www.bioinformatics.babraham.ac.uk/projects/fastqc/) and low-quality reads (Phred quality score < 15 and read length < 30 bp) were discarded using Fastp v0.23.4 (43). Trimmed reads were pseudo-aligned against the European seabass reference transcriptome (seabass_V1.0 Annotation release 105) using Kallisto v0.46.1 (44). Transcript level expression was imported to R v4.2.1 using R/tximport v1.26.1. Principal component analysis (PCA) was performed to visualize the spatial distribution of the dietary groups at each sampling time. The R/DESeq2 package v1.38.3 (45) was used for differential expression gene analysis. Three comparisons were made for each time point post-infection (24 and 48 hpi), with CTRL, MET2 and MET3 groups being contrasted to CTRL before infection (i.e., CTRL 0h), used as a common reference. Genes with False Discovery Rate adjusted *p*-values ≤ 0.01 and an absolute Log2 Fold Change (Log2FC) ≥ 2 in relation to the CTRL 0h group were considered differentially expressed genes (DEGs).

#### 2.6.2 Gene Ontology functional enrichment

Gene Ontology (GO) enrichment analyses were performed using the g:GOSt tool from the g:Profiler web server (https://biit.cs.ut.ee/gprofiler/gost). Enriched GO terms associated with biological processes, cellular components and molecular function were detected using Benjamini-Hochberg FDR for simple multiple testing correction, considering a cut-off FDR *p*-value < 0.05. Bubble plots of the enriched GO terms were built using the R/ggplot2 package v3.5.0.

Whenever the gene name within a specific enriched GO term was unavailable, its peptide sequence was generated using the Ensemble BioMart tool (https://www.ensembl.org/biomart/martview/bed4134e88e4bdcb0cb8e8922cf2c55d). This sequence was then aligned against the NCBI (National Center for Biotechnology Information) database using the protein-protein BLAST (Basic Local Alignment Search Tool) tool (BLASTp, https://blast.ncbi.nlm.nih.gov/Blast.cgi?PROGRAM=blastp&PAGE_TYPE=BlastSearch&LINK_LOC=blasthome). Relevant information, including the best-matching protein sequences, query cover, total score and maximum score, was collected from the BLAST results (**Supplementary File S2**).

### 2.7 Data analysis

Except for the RNA-seq, the IBM SPSS v29.0 Statistics for Windows was used to test data for normality and homogeneity of variances through the Shapiro-Wilk and Levene’s tests, respectively. Whenever necessary, outliers were removed and data was transformed. A one-way ANOVA was performed to investigate the effects of the diets on the fish’s immune status after the 4 weeks of the feeding trial and a two-way ANOVA was performed to explore the effects of diet and time on the early response to *T. maritimum*. Whenever diet or time was significant, the Tukey *post hoc* test was performed to identify differences among groups within the variables. The Chi-square test was performed to understand if there was a relationship between dietary treatment and cumulative mortality. A multivariate canonical discriminant analysis (DA) was also performed using XLSTAT v2023.1.2. Discriminatory effectiveness was assessed using Wilk’s Lambda test and the distance between group centroids was measured using the Mahalanobis distance. Fisher’s F statistic was applied to infer significance (**Supplementary File S3**). For all the tests, 95% CI was used, giving a probability level of 0.05. Data are presented as means ± standard deviation (mean ± SD).

## 3 Results

### 3.1 Cumulative mortality

**Figure 1:**
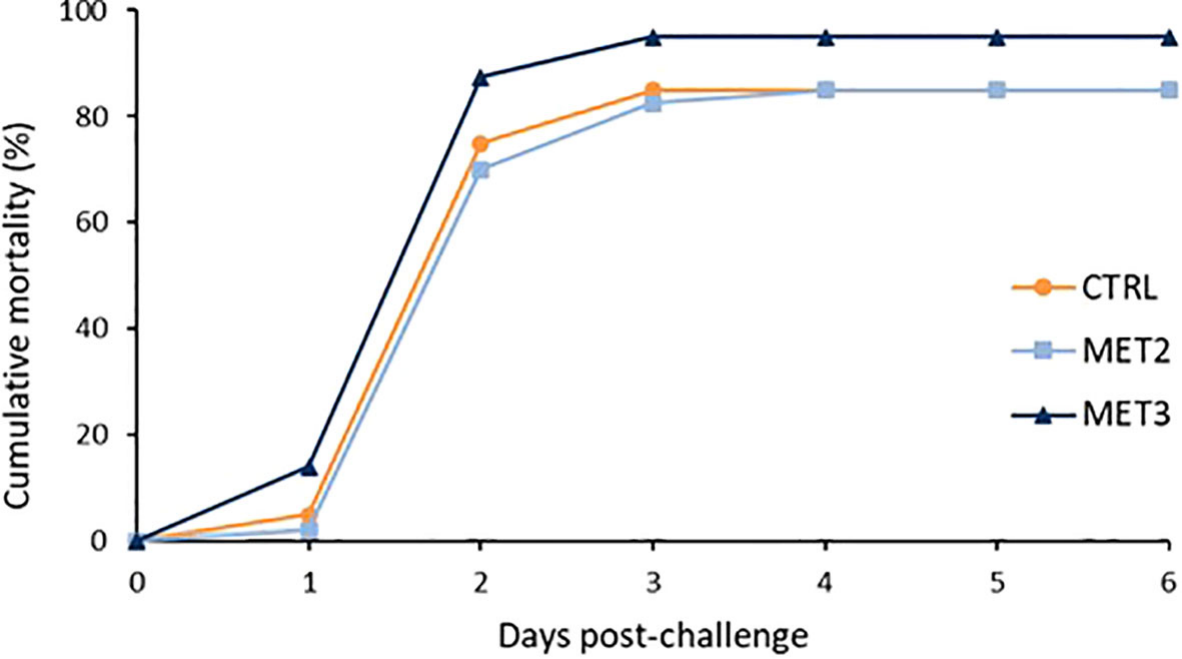
Cumulative mortality of European seabass bath infected with *T. maritimum* (1 × 10^6^ CFU ml^-1^) after 4 weeks of feeding with CTRL (•), MET2 (▪) or MET3 (▴) diets (n=40). Sham-infected fish are not presented for the clarity of the graph.

The mortality profiles across the different dietary groups were similar. Regardless of dietary group, the first deaths were registered one day after the infection and the cumulative rate peaked two days post-infection (**Figure 1**). At the end of the mortality trial, the MET3 group displayed a higher cumulative mortality rate (95%) compared to the other two dietary groups (85%), although not statistically significant (*χ^2^* = 0.274). As expected, no mortality was recorded in sham-infected fish.

### 3.2 Immune status

Except for hemoglobin levels, which decreased with increasing dietary methionine supplementation, no significant differences were observed among dietary groups after the 4 weeks feeding trial in any other hematologic parameter (**Supplementary File S1**, **Supplementary Table S2**). Similarly, no significant differences were observed at the end of the feeding period in peripheral blood leucocyte populations (**Supplementary File S1**, **Supplementary Table S3**), skin mucus immune parameters (**Supplementary File S1**, **Supplementary Table S4**), intestinal antioxidant capacity (**Supplementary File S1**, **Supplementary Table S5**), or gene expression in the head kidney (**Supplementary File S1**, **Supplementary Table S6**).

### 3.3 Inflammatory response

**Figure 2:**
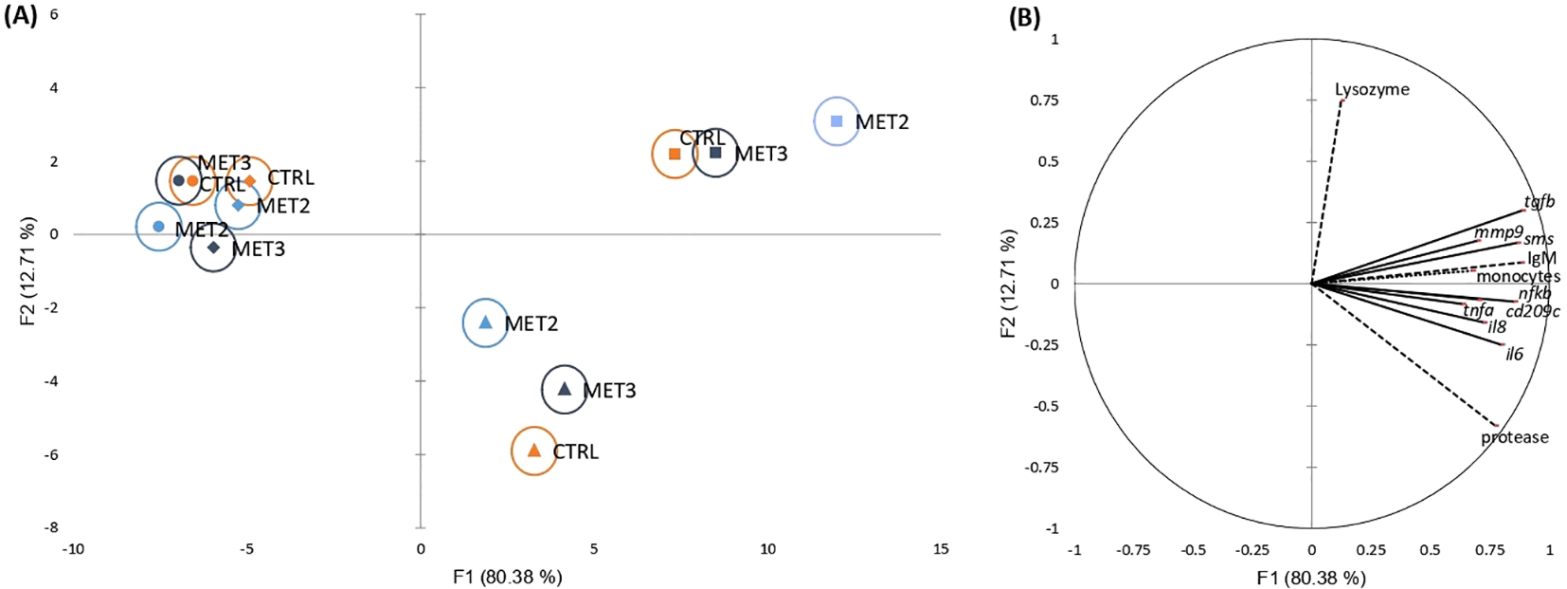
Canonical discriminant analysis of European seabass peripheral leucocyte counts (……), skin mucus immune response (---) and head kidney gene expression (—) before infection (•) and at 4 (♦), 24 (▴) and 48 hpi (▪). **(A)** Canonical discriminant scores of each group. A small circle mark represents group centroids, whereas ellipses indicate data distribution per group. **(B)** Correlation variables/factors (factor loads) for two main discriminant functions (F1 and F2).

The activation of defense mechanisms in response to *T. maritimum* was evident across all dietary groups (**Supplementary File S1**, **Supplementary Tables S7**, **S8**, **S9** and **S11**). To better understand the immune response patterns across treatments, all analyzed parameters were integrated into a single dataset and their overall importance was examined using a discriminant analysis (DA). The Wilk’s Lambda test was highly significant (*p* < 0.0001), indicating the significance of the DA model (**Supplementary File S3**). The first two discriminant functions explained 93.09% of the total dataset variation (**Figure 2**; F1 80.38% and F2 12.71%).

The separation of pre- and post-infection groups was positively influenced by peripheral monocyte trafficking, skin mucus IgM levels and protease activity, and the gene expression levels of transforming growth factor-beta (*tgfb*), spermine synthase (*sms*), nuclear factor kappa B (*nfkb*), interleukins 6 and 8 (*il6* and *il8*, respectively), cluster of differentiation 209 antigen-like protein C (*cd209c*), matrix metallopeptidase 9 (*mmp9*) and tumor necrosis factor-alpha (*tnfa*) in the head kidney (**Figure 2**). Additionally, the discrimination of 24 hpi from other time points was positively affected by protease activity and *il6* expression and negatively influenced by lysozyme activity. Conversely, the 48 hpi discrimination was positively associated with lysozyme activity and *tgfb* expression and negatively associated with protease activity.

Regarding the effects exerted by dietary methionine supplementation on the fish inflammatory response, the clearest discrimination among dietary groups was observed at the later time points post-infection (i.e., 24 and 48 hpi). The MET2-fed group displayed the most moderated immune response at 24 hpi, being less influenced by protease activity and inflammatory-related genes than the other groups. However, by 48 hpi, its clear separation from the other dietary groups on the x-axis suggested a heightened immune response (**Figure 2**).

**Figure 3:**
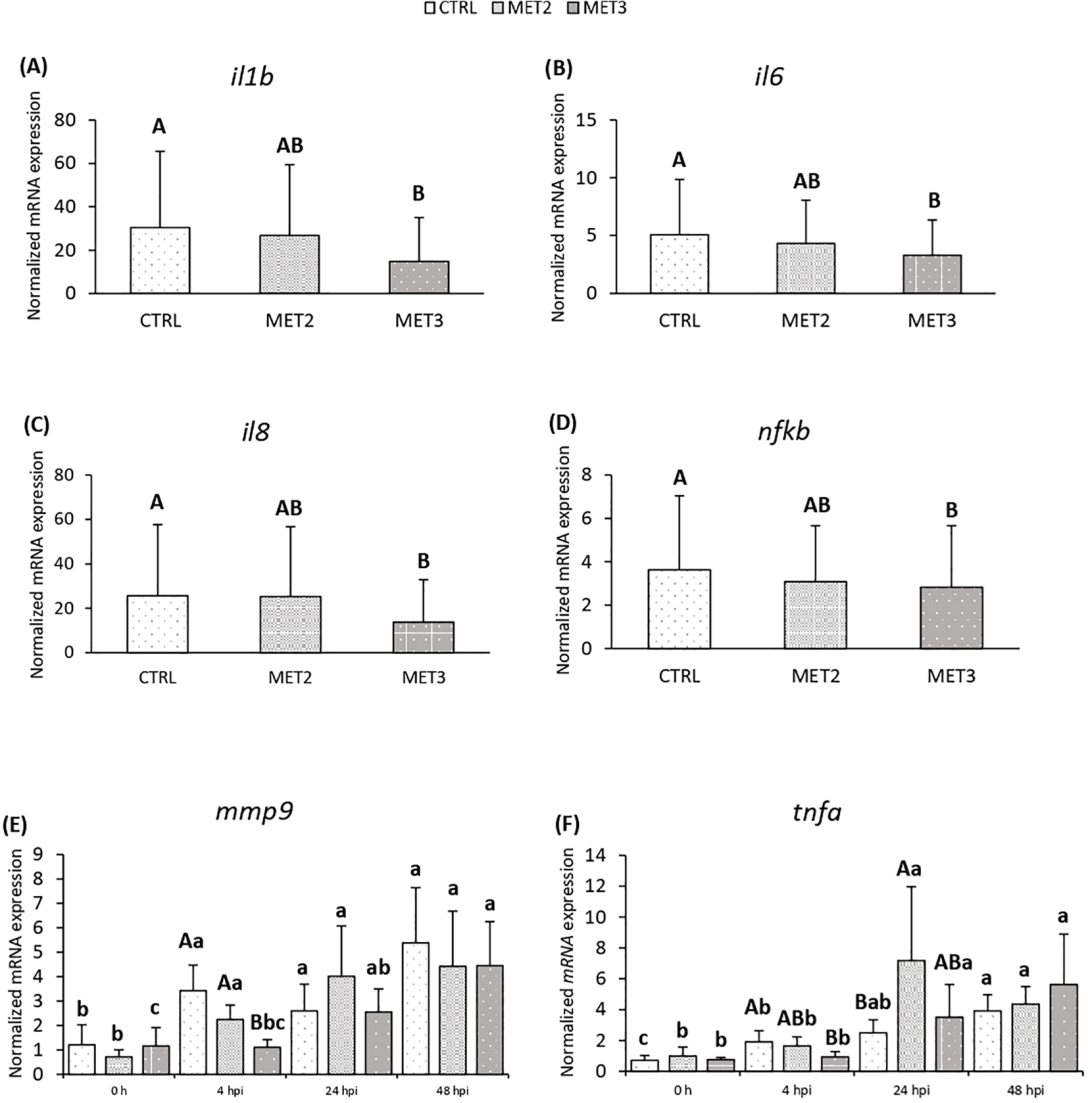
Expression of **(A)** *il1b*, **(B)** *il6*, **(C)** *il8*, **(D)** *nfkb*, **(E)** *mmp9* and **(F)** *tnfa* in the head kidney of European seabass fed dietary treatments for 4 weeks (0 h), at 4, 24 and 48 hours post-infection. Data are expressed as mean ± SD (n=24 per treatment for **(A-D)**, n=6 per treatment for **(E, F)**. Different capital letters stand for differences among dietary treatments and different lowercase letters indicate differences among times (Two-way ANOVA; *p* ≤ 0.05).

Additionally, the expression of proinflammatory mediators interleukin 1-beta (*il1b*), *il6*, *il8* and *nfkb* in the head kidney decreased with increasing methionine intake, with significantly lower levels observed in fish fed the MET3 diet compared to the CTRL group, regardless of sampling point (**Figures 3A-D**). At 4 hpi, *mmp9* expression was also reduced in the MET3 group compared to the other dietary groups, although this difference was no longer evident at later time points (**Figure 3E**). Similarly, at 4 hpi, *tnfa* expression decreased with the higher methionine supplementation, being significantly lower in MET3-fed fish than in the CTRL group. At 24 hpi, however, the highest *tnfa* expression level was observed in the MET2 group, which, although not significantly different from the MET3 group, was higher than in the CTRL group (**Figure 3F**).

### 3.4 Skin tissue RNA-seq principal component analysis

**Figure 4:**
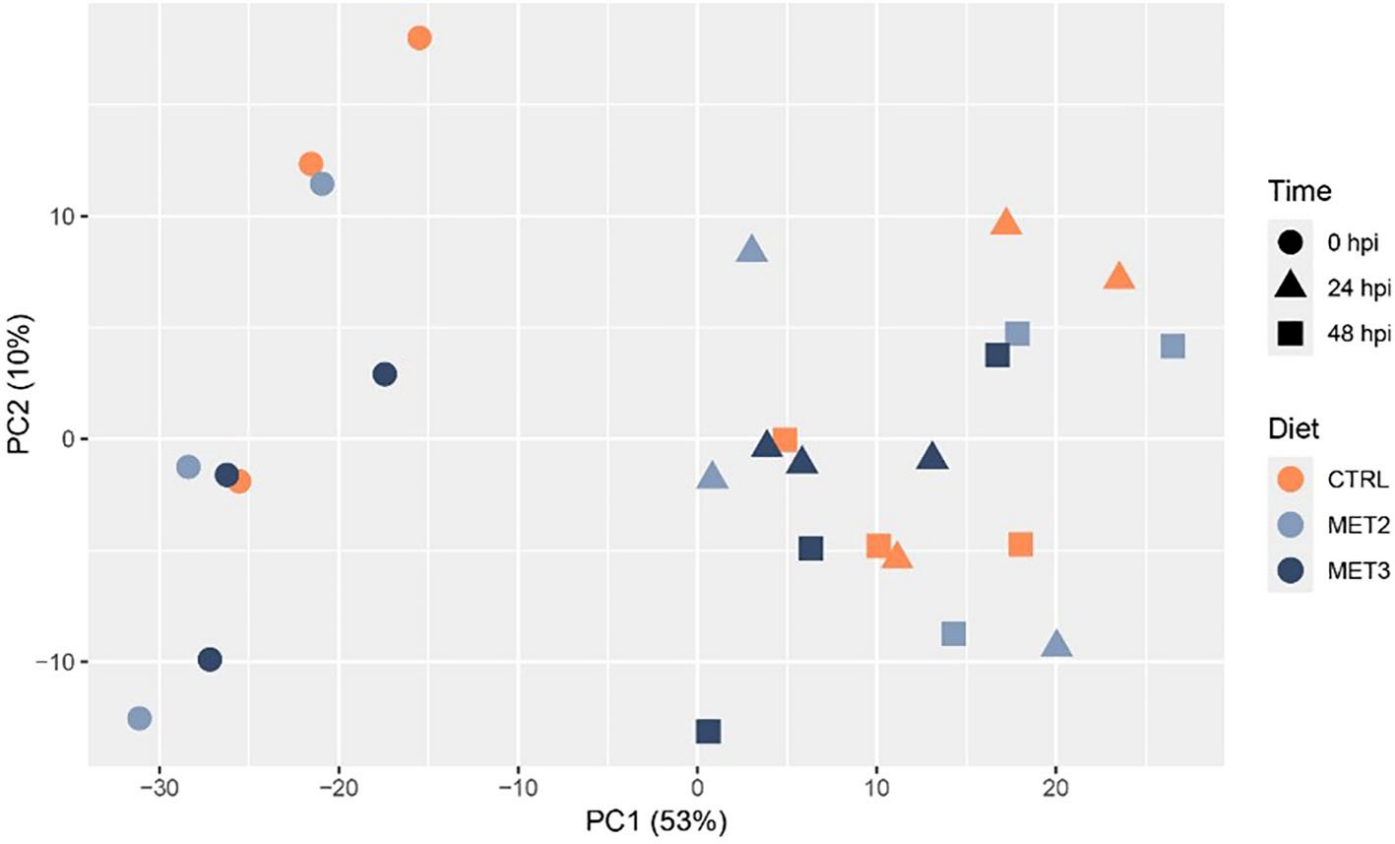
Principal component analysis showing the clustering of RNA-seq data from the skin of European seabass-fed experimental diets (CTRL, MET2 and MET3) during 4 weeks, before (0 hpi) and after bath-challenge (24 and 48 hpi) with *T. maritimum*.

Skin tissue samples collected before infection (0 hpi) and at 24 and 48 hpi were RNA sequenced. Principal component analysis (PCA) revealed a clear separation of infected and non-infected fish. However, no discernible separation was observed between 24 and 48 hpi samples and fish fed different dietary treatments overlapped within time points (**Figure 4**).

### 3.5 Differential expression analysis

**Figure 5:**
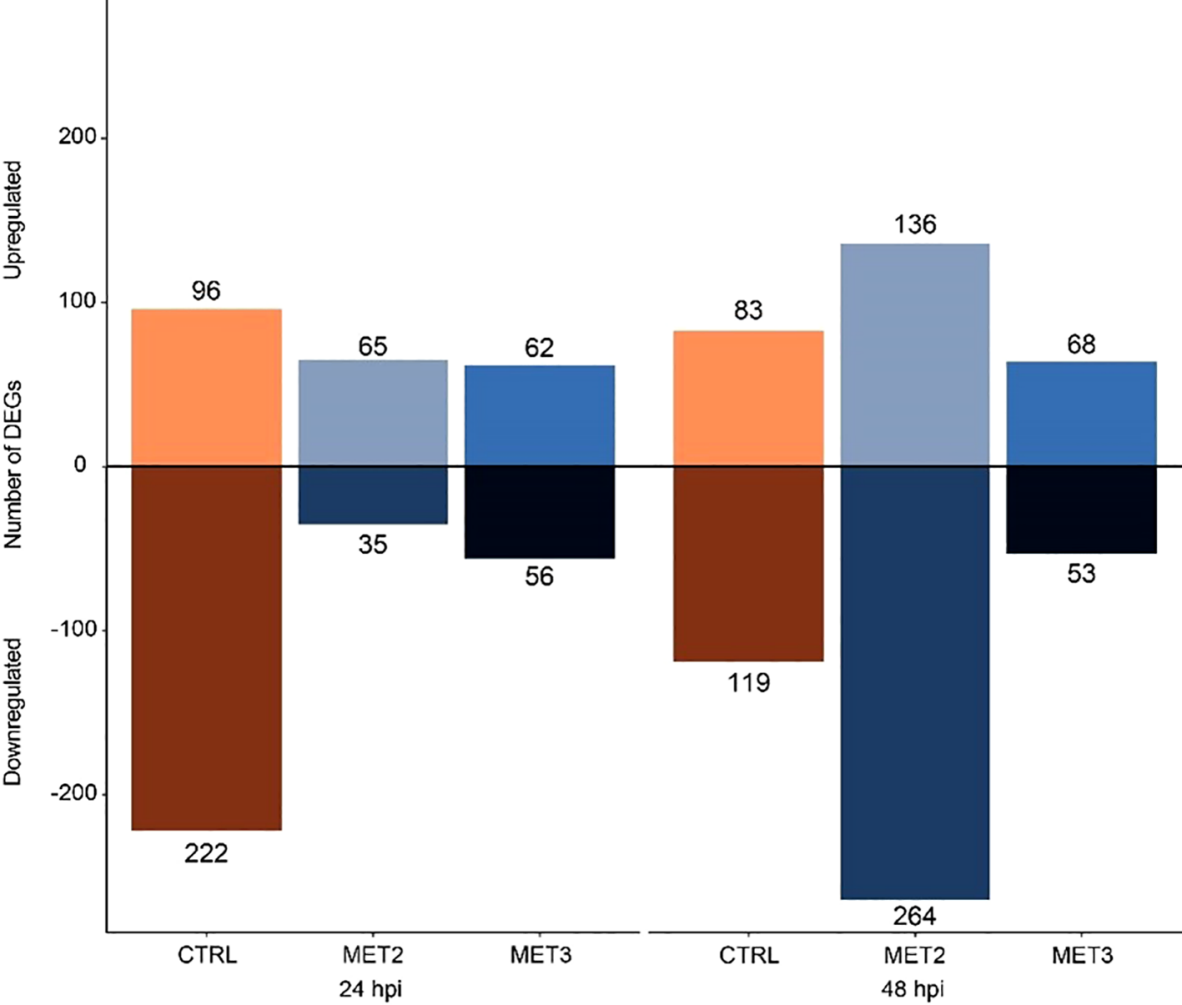
Diverted stacked bar chart showing differentially expressed genes (padj < 0.01 and (Log2FC) ≥ |2|) in the skin of European seabass-fed experimental diets (CTRL, MET2 and MET3) during 4 weeks, at 24- and 48-hours post-infection with *T. maritimum*.

Differential expression analysis was performed between the CTRL group pre-infection and the infected samples. The analysis revealed a total of 318, 100, and 118 DEGs for CTRL, MET2, and MET3, respectively, at 24 hpi. At 48 hpi, the number of DEGs decreased to 202 in the CTRL group, substantially increased to 400 in the MET2 group and remained similar in the MET3 group, with 121 DEGs (**Figure 5**; **Supplementary File S4**). Different patterns in the number of up- and downregulated genes were observed across the different dietary groups at the different time points analyzed. For instance, at 24 hpi, more genes were downregulated (222) than upregulated (96) in the CTRL group, whereas the methionine-supplemented groups had a greater number of upregulated genes than downregulated (65 and 62 upregulated DEGs against 35 and 56 downregulated, in the MET2 and MET3 groups, respectively). At 48 hpi, although the CTRL and MET3 groups kept the pattern of up- and downregulated genes observed in the previous timepoint, i.e., the CTRL group remained with a higher number of downregulated genes (119) than upregulated (83), and the MET3 group had more positively regulated genes (68) than negatively (53), an inverse pattern was observed in the MET2 dietary group, which, at this timepoint, had more downregulated (264) than upregulated genes (136) (**Figure 5**).

Serum amyloid A (SAA) and complement component 7b (C7b) were among the immune-related DEGs upregulated across all dietary groups at both times post-infection. On the opposite, at 24 hpi, the pro-inflammatory IL1β and IL8 were exclusively upregulated in the CTRL group. By 48 hpi, both interleukins were upregulated in the MET2 group, while neither interleukin was upregulated in the MET3 group at any time. Moreover, in the MET3 group at 24 hpi, the heat shock protein beta-11 (HSPβ11) was significantly downregulated (log2FC ≈ -26, *p*-value = 9.32E-05), marking it as the most suppressed gene in the entire study (**Supplementary File S4**).

### 3.6 Enrichment analysis

**Figure 6:**
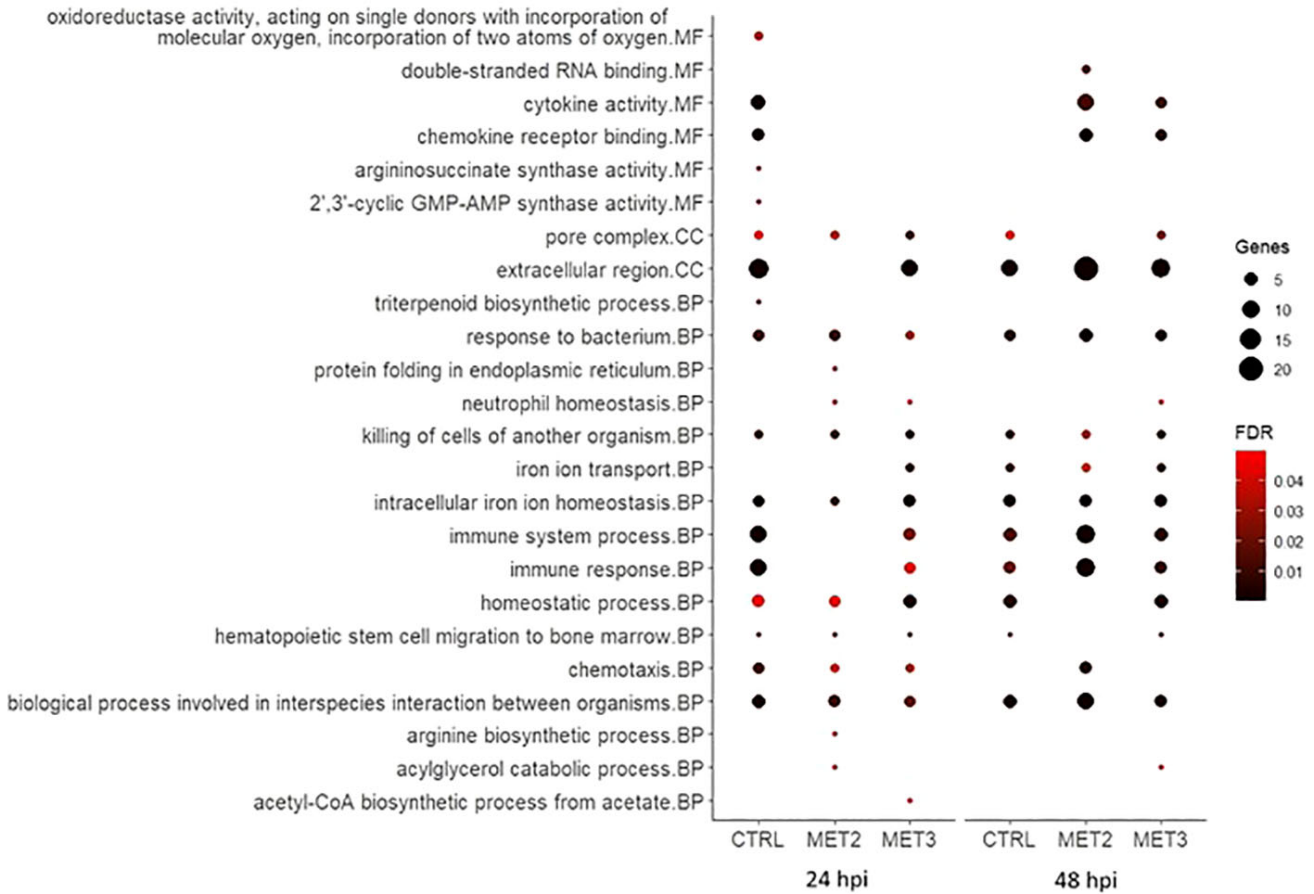
Bubble chart of the gene ontology (GO) enrichment analysis in upregulated DEGs in the skin of European seabass-fed experimental diets (CTRL, MET2 and MET3) during 4 weeks, 24- and 48-hours post-infection with *T. maritimum*. GO terms are categorized into Molecular Function (MF), Cellular Component (CC), and Biological Process (BP).

**Figure 7:**
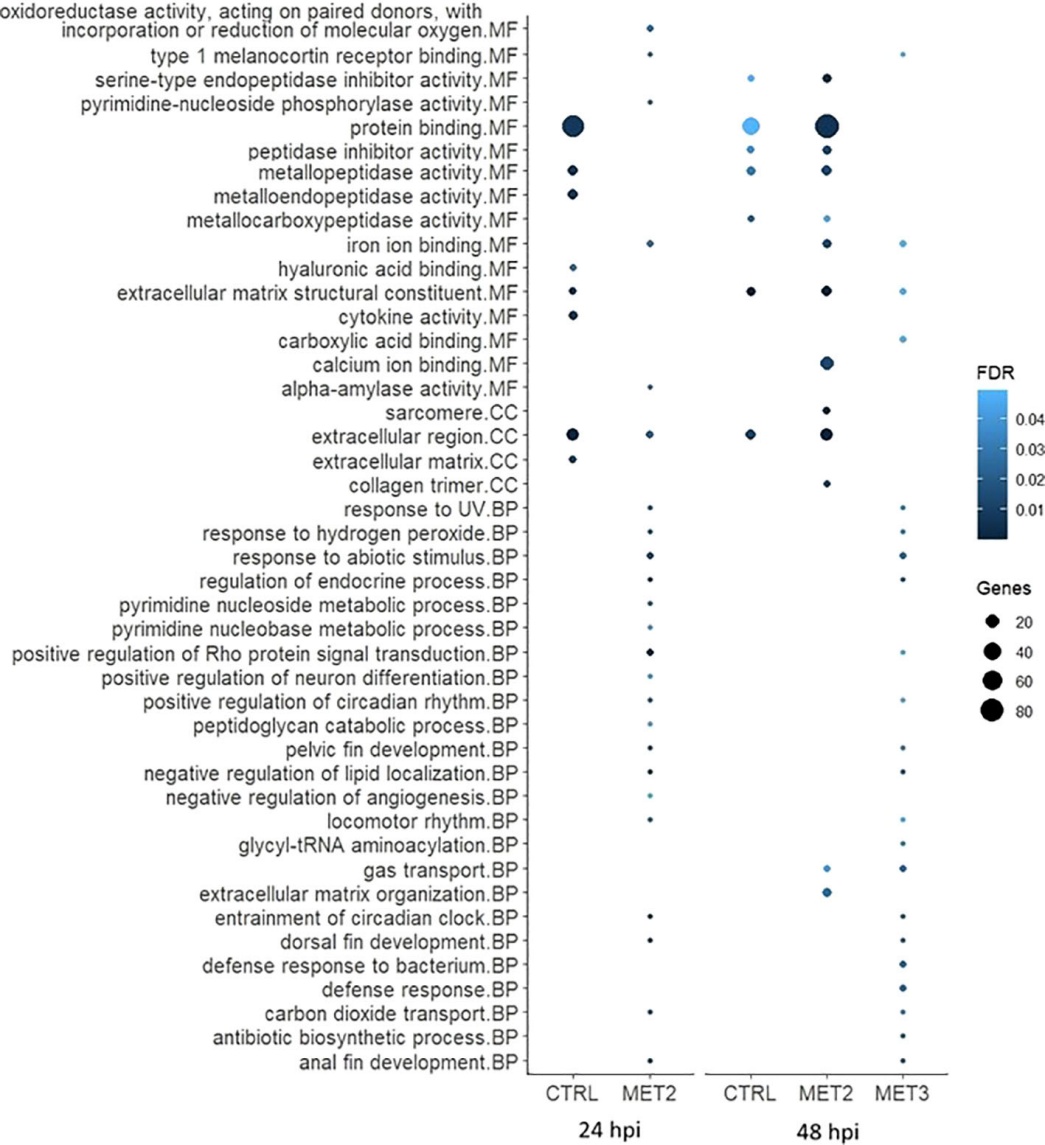
Bubble chart of the gene ontology (GO) enrichment analysis in downregulated DEGs in the skin of European seabass-fed experimental diets (CTRL, MET2 and MET3) during 4 weeks, 24- and 48-hours post-infection with *T. maritimum*. GO terms are categorized into Molecular Function (MF), Cellular Component (CC), and Biological Process (BP).

To further explore DEGs functional implications, Gene Ontology (GO) enrichment analyses were conducted on significantly up- and downregulated DEGs for each comparison (**Figures 6**, **7**; **Supplementary File S5**). The enriched pathways varied across time and dietary groups.

At 24 hpi, immune-related processes such as “cytokine activity”, “chemokine activity”, “response to bacterium”, “killing of cells of another organism”, “intracellular iron ion homeostasis”, “immune system process”, “immune response”, “chemotaxis” and “biological process involved in interspecies interaction between organisms” were, unsurprisingly, enriched amongst upregulated genes in the CTRL group. The interleukins 1-beta and 8 (IL1β and IL8), chemokine (C-X-C motif) ligand 18b (CXCL18b), chemokine (C-C motif) ligand 19 (CCL19), granulocyte colony-stimulating factor (G-CSF), hepcidin types 1 and 2 (HAMP1 and HAMP2, respectively) were among the upregulated transcripts with obvious immune functions (**Supplementary File S5**). At 48 hpi, the terms “cytokine activity”, “chemokine activity” and “chemotaxis” were no longer enriched, and the number of genes involved in “immune system process” and “immune response” decreased (**Figure 6**), with some of the transcripts above mentioned, such as IL8, CXCL18b and G-CSF, no longer being upregulated in this dietary group (**Supplementary File S5**).

In contrast, in the MET2 group at 24 hpi, the most enriched processes amongst upregulated DEGs included “response to bacterium”, “killing of cells of another organism” and “intracellular iron ion homeostasis”. Moreover, terms such as “cytokine activity”, “chemokine activity”, “immune system process” and “immune response” became enriched only at 48 hpi, but with a higher number of DEGs compared to the observed in the CTRL group. Furthermore, “chemotaxis” and “biological process involved in interspecies interaction between organisms” displayed lower FDR values at 48 hpi compared to the observed at 24 hpi and to the CTRL group at 48 hpi (**Figure 6**). Among the immune-related transcripts upregulated in this dietary group at both time points some genes stand out, namely the antimicrobial peptide NK-lysin-like (NK-lysin), SAA and transferrin (TFA) (**Supplementary File S5**).

In the MET3 group at 24 hpi, the most enriched terms for upregulated genes were predominantly associated with iron metabolism and homeostasis, including processes such as “iron ion transport”, “intracellular iron ion homeostasis” and “homeostatic process”. Additionally, “pore complex” and “killing cells of another organism” were enriched. Although terms such as “immune system process”, “immune response”, “chemotaxis” and “biological process involved in interspecies interaction between organisms” were also enriched among the upregulated DEGs, they exhibited higher FDR and lower number of DEGs compared to the CTRL at the same time point. Similar to the MET2 group, these processes’ enrichment increased at 48 hpi in the MET3 group, though to a lesser extent. Additionally, the terms “cytokine activity” and “chemokine activity” were enriched at 48 hpi, although less pronouncedly than in the MET2 group (**Figure 6**).

The processes among downregulated DEGs in the CTRL group at 24 hpi, included “protein binding”, “metallopeptidase activity”, “metalloendopeptidase activity”, “extracellular matrix structural constituent”, “cytokine activity”, “extracellular region” and “extracellular matrix”. The interleukins 17C, 17F and 8 (IL17C, IL17F and IL8, respectively) and the chemokines (C-X-C motif) ligand 12b and 19 (CXCL12b and CXCL19, respectively) were among the downregulated transcripts with recognized immune roles in this group at that time (**Supplementary File S5**). At 48 hpi, most of these terms were either less enriched or not enriched, except for “extracellular matrix structural constituent” which remained unaltered (**Figure 7**).

In the MET2 group at 24 hpi, despite the numerous GO terms among downregulated DEGs, many included only a single gene, often common to multiple processes (**Supplementary File S5**). The most enriched pathway was the “positive regulation of Rho protein signal transduction”. At 48 hpi, all the terms enriched in the CTRL group were also enriched in the MET2 group, displaying even greater enrichment. Additionally, terms not enriched in the CTRL group, such as “sarcomere”, “collagen trimer”, “calcium ion binding” and “iron ion binding” were highly enriched in the MET2 group (**Figure 7**).

In the MET3 group at 24 hpi, downregulated DEGs did not combine into discernible GO terms. At 48 hpi, similarly to that observed in the MET2 group at 24 hpi, most terms featured only one gene, often shared across different processes. Comparable to the MET2 group, the pathways “entrainment of circadian clock”, “regulation of endocrine process”, “negative regulation of lipid localization”, “anal fin development” and “dorsal fin development” were enriched in the MET3 group. Additionally, terms such as “gas transport”, “response to abiotic stimulus”, “defense response to bacterium” and “defense response” ranked among the most enriched processes among downregulated DEGs (**Figure 7**).

## 4 Discussion

Enhanced methionine supplementation beyond basic requirements has been proven to improve both immune and antioxidant systems, besides conferring benefits for stress and disease resistance (46). However, its optimal intake thresholds are narrower than other amino acids, underscoring the importance of carefully selecting its supplementation levels in feed formulations (26). Considering the positive outcomes observed in our prior studies regarding the impact of methionine supplementation on European seabass immune status and innate immune responses to inflammation (17, 18), the present study aimed to investigate supplementation levels exceeding those previously tested. Specifically, it aimed to assess the impact of these elevated dietary methionine supplementation levels on the immune status and early immune response of European seabass against *T. maritimum*, particularly focusing on mucosal immune responses.

Although few differences were observed regarding the fish immune status among dietary treatments following the 4 weeks of feeding, increased methionine intake was associated with decreased hemoglobin levels. This pattern aligns with previous studies in fish, where surpassing a certain threshold of methionine intake led to a decline in hemoglobin levels. For instance, Liao, Ren (47) observed a decrease in hemoglobin in *Megalobrama amblycephala* fed diets containing methionine above 10 g kg^-1^ dry diet. Similarly, in the study by Elmada, Huang (16), the maximum hemoglobin level was observed in *Pelteobargrus fulvidraco* fed diets containing 9.7 and 11.8 g kg^-1^, decreasing thereafter with higher dietary methionine content. Notwithstanding, the concentration of methionine in the lowest supplemented diet employed in our study (18.5 g/kg) exceeded the highest concentration used in these two studies.

The decrease in hemoglobin levels associated with excess methionine intake has also been observed in other animals, such as rats (26) and chickens (48). In mammals, these alterations in the hematological profile have been linked to the accumulation of methemoglobin and the formation of Heinz-body in erythrocytes, leading to hemolytic anemia (26). Although the impact of methionine on the number of RBC was not statically significant in the present study, there was a tendency toward a decrease in RBC numbers as methionine intake increased. Nevertheless, despite the consistent reporting of these alterations in various studies, the mechanisms underlying the impact of methionine on the hematological profile, particularly in fish, remain poorly understood and warrant further investigation.

Despite the reduction in hemoglobin levels linked to higher methionine consumption, no significant changes were observed in the overall immune status of the fish. Based on prior studies in seabass, which indicated an enhancement in the immune status with increasing methionine intake (18–21), similar outcomes were anticipated in the present study. Alternatively, a potential detrimental effect due to excessive methionine supplementation was also considered. Surprisingly, while immune status remained unaffected, methionine intake significantly modulated the immune response to *T. maritimum*, as detailed below.

Regardless of dietary treatment, the bath challenge with *T. maritimum* triggered a systemic response, with infected fish presenting monocytosis, along with the activation of several defense mechanisms in the skin mucus (e.g., proteases and lysozyme) and upregulation of proinflammatory cytokines (e.g., IL8 and TNFα) in the head kidney. At 24 hpi, the immune response was characterized by an increase in *il6* mRNA expression, whereas the response at 48 hpi was marked by elevated expression of *tgfb*. During acute inflammation, IL6 promotes monocyte recruitment over neutrophils, facilitating the resolution of inflammation and the initiation of the immune response (49). On the other hand, the upregulation of TGF-β at 48 hpi is rather associated with inflammation control and tissue repair. At high concentrations, TGF-β exhibits anti-inflammatory properties and promotes fibrosis and extracellular matrix production by fibroblasts (50). Given that tenacibaculosis is an ulcerative disease, the increased expression of TGF-β likely reflects tissue damage and repair processes.

Although all dietary groups responded to the bacteria, the mRNA expression levels of proinflammatory mediators, including *il1b*, *il6*, *il8* and *nfkb*, were significantly reduced in the head kidney of fish fed higher methionine supplementation levels in a dose-dependent manner. NF-κB is a crucial transcriptional factor in initiating the host inflammatory responses to pathogen invasion, orchestrating key processes such as DNA transcription, proinflammatory cytokine production, leucocyte recruitment and cell survival (51). While excessive inflammation can harm the host, a robust inflammatory response is essential for effective pathogen defense in the early stages of infection (52).

The attenuation of the inflammatory response associated with increased methionine supply has been documented in previous studies (53). For instance, an *in vitro* study by Ji, Xu (54), demonstrated that methionine inhibits the mitogen-activated protein kinase (MAPK) pathway by altering DNA methylation. This inhibition leads to a decrease in the abundance of proinflammatory cytokines and chemokines, such as IL6 and TNFα, thereby dampening the typically proinflammatory response triggered by lipopolysaccharide (LPS), the major component of the outer membrane of Gram-negative bacteria (54–56). Furthermore, Wang, Liang (57) reported that methionine reduced hepatic levels of an oxidative stress biomarker, by stimulating glutathione S-transferase (GST) activity, which inhibited NF-κB activation and decreased the expression of inflammatory mediators like IL1β and TNFα. Similarly, Hasegawa, Ichiyama (58) observed that cysteine, a metabolite of methionine, exhibited a great inhibitory effect on NF-κB activation, attributed to its antioxidant properties stemming from its sulfhydryl group. Overall, these findings suggest that methionine may dampen the immune response through multiple pathways, including DNA methylation, modulation of phosphorylation states and enhancement of antioxidant defenses, either directly or through its metabolite cysteine.

Considering that the teleost skin serves as the initial barrier against pathogen invasion and given its importance in the initiation of the immune response against this specific pathogen (59), the present study used transcriptomic profiling to investigate the impacts of dietary methionine supplementation on European seabass skin immune response against a waterborne bacterium. Along with the activation of the immune defense mechanisms mentioned above, the clear separation between pre- and post-infection groups in the PCA plot and the nature of enriched GO terms indicated the activation of immune mechanisms in the skin, irrespective of dietary treatments. While prior studies have already described the triggering of a localized immune response following bath infection with *T. maritimum* (42, 60), the magnitude of the inflammatory response and the immune mechanisms activated at each time point appeared to be influenced by the level of methionine intake.

Looking first at the response of CTRL-fed fish, at 24 hpi it was observed the strongest immune response at the skin level, as indicated by the greatest distance from the other dietary groups in the PCA plot and the highest number of DEGs and lower FDR of enriched immune-related pathways (e.g., “cytokine activity”, “immune response” and “immune system process”). However, the inflammatory response appeared to diminish over time, as evidenced by the lack of enrichment of some immune-related pathways at 48 hpi (e.g., “cytokine activity” and “chemokine receptor binding”) and the lower number of genes and higher FDR of others (e.g., “immune system process” and “immune response”). These transcriptomic findings from the skin were consistent with those obtained from the DA, where at 24 hpi, the CTRL group was the most positively influenced by *il6* and protease activity compared to other groups, while at 48 hpi, it was the least influenced group by all the immune-related genes. Moreover, the skin immune response pattern observed in the present study closely resembled findings reported by Ferreira, Peixoto (42), where *T. maritimum* bath challenge induced a significant surge in the expression of *il1b* and *il8* in European seabass skin at 24 hpi, followed by a return to baseline levels at 48 hpi. IL1β and IL8 play pivotal roles in the host response to bacteria, with the former initiating the inflammatory response and the latter activating and recruiting neutrophils and T cells to the inflammatory sites (61, 62). Additionally, CXCL18B, CCL19 and G-CSF were among the DEGs involved in immune-related pathways in the CTRL-fed group of the present study. CXCL18b and CCL19 mediate immune cell trafficking (63), whereas G-CSF stimulates the proliferation, survival and differentiation of immune cells (64). Furthermore, both studies noted a concurrent increase in the expression of genes related to iron metabolism at 24 and 48 hpi (42). Alongside the upregulation of HAMP1 observed by Ferreira, Peixoto (42), the increased expression of hepcidin-like and HAMP2 was responsible for the significant enrichment of the term “intracellular iron ion homeostasis” in the present study. While HAMP2 is recognized by its direct antimicrobial activity, HAMP1 regulates iron homeostasis by inhibiting the iron exporter ferroportin (65), thereby retaining iron and depriving bacteria of this nutrient essential for growth and survival (66).

Despite the activation of all aforementioned defense mechanisms, “cytokine activity” was enriched among downregulated genes in the CTRL-fed group at 24 hpi. While the inhibition of proinflammatory interleukins (e.g., IL17C, IL17F and IL8) and CXC chemokines (e.g., CXCL12b and CXCL19) could be a mechanism to prevent an excessive inflammatory response that could potentially harm the host’s own cells (62), it might rather indicate a suppression of the host defense mechanisms induced by *T. maritimum* secretory/excretory products, rendering fish more vulnerable to infection (33, 67). This suppression could help explain the high mortality rate observed in this dietary group by the end of the trial (85%).

The modulation of the host regulatory GTPases of the Rho family is another well-known strategy employed by many bacterial pathogens, including those affecting fish (68, 69), to manipulate the host immune response. These proteins play a crucial role in regulating the actin cytoskeleton, which is essential for cellular integrity and host-pathogen interactions (69, 70). Through the release of toxins and enzymes, pathogens disrupt the regulatory roles of Rho GTPases, altering the normal functioning of host signaling pathways and creating an environment conducive to their proliferation (69, 71). Although there is a lack of specific information concerning *T. maritimum* in this regard, several studies exploring the array of enzymes and toxins produced by this species to promote colonization and infection have been published (72, 73). Therefore, the modulation of host Rho proteins by *T. maritimum* remains a plausible possibility. In the present study, the “positive regulation of Rho protein signal transduction” pathway had the lowest FDR value among the downregulated DEGs in the MET2 group at 24 hpi.

Moreover, the MET2-fed group appeared to display a dampened immune response at 24 hpi compared to their counterparts fed the CTRL diet, characterized by a reduced number and lower expression of upregulated DEGs directly related to immunity. Indeed, despite an increased expression of some innate immune-related transcripts, including the NK-lysin, SAA and TFA, which are known for their effective antibacterial activity (74–77), immune cells recruitment to the inflammatory sites (78, 79) and limitation of iron availability to bacteria (80, 81), respectively, the MET2-fed group did not experience upregulation of some of the proinflammatory mediators observed in those fish fed the CTRL diet at the same time point (e.g., IL1β, IL8, G-CSF and CXCL18b). Although less pronounced inflammatory responses are generally associated with more disease-resistant phenotypes (82), in the present study, the initially attenuated immune response of the MET2 group at 24 hpi was replaced by a heightened immune response at 48 hpi, characterized by the activation of immunological mechanisms identical to the ones activated in the CTRL group at 24 hpi but on a larger scale. The aggravated inflammatory response might stem from the advanced colonization of fish skin by *T. maritimum*. Supporting this hypothesis is the downregulation of pathways such as “sarcomere” and “collagen trimer” in this group at 48 hpi, indicating a potential impact on the host’s muscle structure or function and wound healing capacity (83, 84). The pathogenesis of *T. maritimum* is a multifactorial process, primarily causing extensive skin damage (33). Therefore, the healing response assumes a central role and wound contraction is part of the process. Contraction helps reduce the size of the damaged tissue, subsequently decreasing the amount of tissue that requires repair (83). There is strong evidence that this bacterium produces tissue-destroying enzymes (85) and probably toxins that could impair the fish skin’s regenerative capacity (33). These adverse effects observed in the skin may extend to other organs, as evidenced by the DA results, which showed a transition from the mildest immune response at 24 hpi to the most intense response at 48 hpi in the MET2 group. Furthermore, the increased influence of TGF-β on the discrimination of this dietary group at 48 hpi might also be linked to an attempt to mitigate tissue damage.

The MET3 was the dietary group with the lowest number of DEGs and minimal variation between 24 and 48 hpi. It seemed to prioritize homeostatic and iron-related pathways over immunological pathways, which, compared to the CTRL group, were less enriched and had fewer number of DEGs. Moreover, at 24 hpi, the heat shock protein beta-11 (HSPβ11) in this dietary group emerged as the most downregulated transcript in the entire study. Heat shock proteins are critical to maintaining cellular homeostasis and protecting cells from diverse stresses, including infection. They are responsible for assembling, folding and translocating other proteins, making them indispensable for sustaining cellular protein homeostasis. In the presence of stressors, they prevent misfolding and aggregation of proteins while facilitating the elimination of the damaged ones (86). Although specific information regarding the roles of HSPβ11in teleost skin is limited (87), its substantial downregulation in the MET3 group upon infection highlights the potential link with the immune response outcome and disease resistance phenotype. The significance of this molecular chaperone becomes particularly pronounced in scenarios involving high methionine intake, as observed at the highest supplementation level in this study (29.2 mg g^-1^). Elevated methionine levels are believed to disrupt protein homeostasis by increasing oxidative stress levels through increased production of reactive oxygen species, impairing the function of protein quality control mechanisms. This disruption leads to the accumulation of misfolded or damaged proteins, contributing to cellular dysfunction (88).

Similar to the MET2 group, neither “cytokine activity” nor “chemokine receptor binding” were enriched at 24 hpi in the MET3 group, gaining significance only at 48 hpi, albeit to a lesser extent. Furthermore, contrary to what was observed in the MET2 group, the increase in enrichment and number of immune-related pathways at 48 hpi was accompanied by the downregulation of other DEGs associated with the immune response, such as “defense response”, “defense response to bacteria” and “antibiotic biosynthetic process”, suggesting an impaired response to the infection (89). The lower expression of proinflammatory mediators observed in the head kidney of fish fed the MET3 diet may contribute to this diminished immune response, indicating a potential deficiency in the immune activation. Although not statistically significant, this dietary group presented greater susceptibility to bacteria since the first 24 hpi, reaching the highest mortality rate (95%) at the end of the feeding trial.

## 5 Conclusion

In summary, while elevated methionine intake was primarily associated with decreased hemoglobin levels, it significantly influenced the immune response to *Tenacibaculum maritimum*. The specific defense mechanisms activated at each time point, as well as their intensity, varied across dietary treatments. These differences were evident not only at a systemic level but also in the transcriptomic profiles of skin tissue, highlighting the link between nutrition and immunity in fish.

## Data Availability

The datasets presented in this study can be found in online repositories. The names of the repository/repositories and accession number(s) can be found below: https://doi.org/10.6084/m9.figshare.27247428.v1.
