# Supplementary material for: Exploring the effects of dietary methionine supplementation on European seabass mucosal immune responses against Tenacibaculum maritimum
Source: Front Immunol. 2025 Jan 22;16:1513516. doi: 10.3389/fimmu.2025.1513516 (PMC11794538; doi:10.3389/fimmu.2025.1513516)
Supplement: Supplementary file 1 [file Table1.docx]

***Supplementary Material***

**Table S1.** Immune-related genes analyzed by Real-Time PCR. AT, Annealing Temperature.

| **Gene** | **Acronym** | **Accession number** | **Efficiency** | **AT (ºC)** | **Amplicon length** | **Primer sequence (5’ – 3’)** |
| --- | --- | --- | --- | --- | --- | --- |
| Elongation factor 1-alpha | *ef1a* | AJ866727.1 | 98.03 | 60 | 144 | F: AACTTCAACGCCCAGGTCAT  R: CTTCTTGCCAGAACGACGGT |
| 40s Ribosomal protein | *40s* | HE978789.1 | 110.17 | 55 | 79 | F: TGATTGTGACAGACCCTCGTG  R: CACAGAGCAATGGTGGGGAT |
| Cluster of differentiation 209 antigen-like protein C | *cd209c* | XM_051406181.1 | 98.04 | 60 | 131 | F: CTCGGTGGCATCCTTCTGAC  R: AGCCAGACAACAACCTGTCC |
| Cluster of differentiation 8 beta | *cd8b* | XM_051415628.1 | 112.75 | 55 | 223 | F: CGGAACCCAAAAGGCCAAAG  R: TAGGCTGTAGATGCAGTGCT |
| Interleukin 1-beta | *il1b* | AJ269472.1 | 112.26 | 57 | 105 | F: AGCGACATGGTGCGATTTCT  R: CTCCTCTGCTGTGCTGATGT |
| Interleukin 6 | *il6* | AM490062.1 | 101.78 | 55 | 81 | F: AGGCACAGAGAACACGTCAAA  R: AAAAGGGTCAGGGCTGTCG |
| Interleukin 8 | *il8* | AM490063.1 | 100.08 | 55 | 140 | F: CGCTGCATCCAAACAGAGAGCAAAC  R: TCGGGGTCCAGGCAAACCTCTT |
| Matrix-metalloproteinase 9 | *mmp9* | FN908863.1 | 96.06 | 57 | 166 | F: TGTGCCACCACAGACAACTT  R: TTCCATCTCCACGTCCCTCA |
| Nuclear factor kappa B | *nfkb* | XM_051395800.1 | 113.28 | 55 | 136 | F: GCTGCGAGAAGAGAGGAAGA  R: GGTGAACTTTAACCGGACGA |
| Spermine synthase | *sms* | XM_051402198.1 | 111.71 | 57 | 132 | F: GCACCTTTGGTTTCTCCTGA  R: AACTCAGTCCCACAGGGTTG |
| Transforming growth factor-beta | *tgfb* | AM421619.1 | 113.54 | 55 | 143 | F: ACCTACATCTGGAACGCTGA  R: TGTTGCCTGCCCACATAGTAG |
| Tumor necrosis factor-alpha | *tnfa* | DQ070246.1 | 92.71 | 55 | 112 | F: AGCCACAGGATCTGGAGCTA  R: GTCCGCTTCTGTAGCTGTCC |

**Table S2.** Hematocrit, hemoglobin, mean corpuscular volume (MCV), mean corpuscular hemoglobin (MCH), mean corpuscular hemoglobin concentration (MCHC), red blood cells (RBC) and white blood cells (WBC) in European seabass fed dietary treatments for 4 weeks. Values are presented as mean ± SD (n=9 per treatment). Different capital letters stand for differences among dietary treatments (One-way ANOVA; *p* ≤ 0.05).

| **Parameters** | | **Dietary treatments** | | | ***p* value** |
| --- | --- | --- | --- | --- | --- |
|  |  | **CTRL** | **MET2** | **MET3** |  |
| **Hematocrit** | **(%)** | 30.38 ± 9.62 | 23.56 ± 7.07 | 26.44 ± 6.25 | ns |
| **Hemoglobin** | **(g dl^-1^)** | 0.94 ± 0.25^A^ | 0.87 ± 0.30^AB^ | 0.59 ± 0.28^B^ | 0.036 |
| **MCV** | **(µm^3^)** | 183.57 ± 58.60 | 170.58 ± 67.89 | 215.54 ± 78.65 | ns |
| **MCH** | **(pg cell^-1^)** | 5.92 ± 2.65 | 5.52 ± 1.30 | 5.79 ± 2.37 | ns |
| **MCHC** | **(g 100 ml^-1^)** | 3.37 ± 1.47 | 2.99 ± 0.58 | 2.19 ± 0.90 | ns |
| **RBC** | **(x10^6^ µl^-1^)** | 1.75 ± 0.56 | 1.64 ± 0.56 | 1.31 ± 0.63 | ns |
| **WBC** | **(x10^4^ µl^-1^)** | 5.10 ± 0.81 | 5.53 ± 1.46 | 5.64 ± 0.93 | ns |

**Table S3.** Absolute values of peripheral blood leucocytes (thrombocytes, lymphocytes, monocytes and neutrophils) of European seabass fed dietary treatments for 4 weeks. Values are presented as means ± SD (n=9 per treatment). P-values from One-way ANOVA; *p* ≤ 0.05.

| **Parameters** | | **Dietary treatments** | | | ***p* value** |
| --- | --- | --- | --- | --- | --- |
|  |  | **CTRL** | **MET2** | **MET3** |  |
| **Thrombocytes** | **(×10^4^ µl^-1^)** | 4.13 ± 0.56 | 4.09 ± 1.15 | 3.82 ± 0.81 | ns |
| **Lymphocytes** |  | 1.10 ± 0.16 | 1.38 ± 0.86 | 1.51 ± 0.29 | ns |
| **Monocytes** |  | 0.06 ± 0.04 | 0.06 ± 0.05 | 0.12 ± 0.05 | ns |
| **Neutrophils** |  | 0.08 ± 0.05 | 0.08 ± 0.04 | 0.10 ± 0.06 | ns |

**Table S4.** Skin mucus immune parameters (peroxidase, protease and lysozyme activities, and IgM levels) of European seabass fed dietary treatments for 4 weeks. Values are presented as means ± SD (n=3 per treatment). P-values from One-way ANOVA; *p* ≤ 0.05.

| **Parameters** | | **Dietary treatments** | | | ***p* value** |
| --- | --- | --- | --- | --- | --- |
|  |  | **CTRL** | **MET2** | **MET3** |  |
| **Peroxidase** | **(units mg^-1^ protein)** | 26.36 ± 10.95 | 23.84 ± 5.46 | 23.57 ± 9.09 | ns |
| **Protease** | **(%)** | 1.38 ± 0.10 | 1.17 ± 0.28 | 0.96 ± 0.23 | ns |
| **Lysozyme** | **(µg mg^-1^ protein)** | 6.02 ± 4.86 | 3.62 ± 1.66 | 6.80 ± 3.91 | ns |
| **IgM** | **(OD)** | 0.02 ± 0.02 | 0.03 ± 0.02 | 0.03 ± 0.01 | ns |

**Table S5.** Intestine oxidative stress biomarkers, catalase activity (CAT) and lipid peroxidation levels (LPO), of European seabass fed dietary treatments for 4 weeks. Values are presented as means ± SD (n=6 per treatment). P-values from One-way ANOVA; *p* ≤ 0.05.

| **Parameters** | | **Dietary treatments** | | | ***p* value** |
| --- | --- | --- | --- | --- | --- |
|  |  | **CTRL** | **MET2** | **MET3** |  |
| **CAT** | **(units mg^-1^ protein)** | 57.21 ± 11.89 | 56.08 ± 3.70 | 61.08 ± 9.91 | ns |
| **LPO** | **(nmol g^-1^ wt)** | 89.35 ± 13.15 | 78.28 ± 14.22 | 77.65 ± 17.11 | ns |

**Table S6.** Quantitative expression of *cd209c*, *cd8b*, *il1b*, *il6*, *il8*, *mmp9*, *nfkb*, *sms*, *tgfb* and *tnfa* in the head kidney of European seabass fed dietary treatments for 4 weeks. Values are presented as means ± SD (n=6 per treatment). P-values from One-way ANOVA; *p* ≤ 0.05.

| **Genes** | **Dietary treatments** | | |  |
| --- | --- | --- | --- | --- |
|  | **CTRL** | **MET2** | **MET3** | ***p* value** |
| ***cd209c*** | 0.77 ± 0.35 | 0.45 ± 0.15 | 2.09 ± 1.80 | ns |
| ***cd8b*** | 1.32 ± 0.97 | 2.43 ± 1.37 | 0.80 ± 0.31 | ns |
| ***il1b*** | 1.40 ± 1.19 | 1.25 ± 0.76 | 2.14 ± 1.75 | ns |
| ***il6*** | 0.85 ± 0.18 | 0.91 ± 0.37 | 0.78 ± 0.24 | ns |
| ***il8*** | 1.35 ± 1.15 | 1.17 ± 0.69 | 0.92 ± 0.46 | ns |
| ***mmp9*** | 1.21 ± 0.82 | 0.72 ± 0.29 | 1.16 ± 0.75 | ns |
| ***nfkb*** | 1.02 ± 0.22 | 0.72 ± 0.21 | 0.66 ± 0.32 | ns |
| ***sms*** | 1.13 ± 0.65 | 0.76 ± 0.26 | 0.59 ± 0.08 | ns |
| ***tgfb*** | 1.05 ± 0.36 | 0.84 ± 0.22 | 0.78 ± 0.47 | ns |
| ***tnfa*** | 0.71 ± 0.32 | 0.99 ± 0.58 | 0.75 ± 0.14 | ns |

**Table S7.** Hematocrit, hemoglobin, mean corpuscular volume (MCV), mean corpuscular hemoglobin (MCH), mean corpuscular hemoglobin concentration (MCHC), red blood cells (RBC) and white blood cells (WBC) absolute numbers in European seabass fed dietary treatments for 4 weeks (0 hpi), at 4, 24 and 48 hours post-infection. Values are presented as mean ± SD (n=9 per treatment). Different capital letters stand for differences among dietary treatments and different lowercase letters indicate differences among times (Two-way ANOVA; *p* ≤ 0.05).

| **Parameters** | | **CTRL** | | |  | |  | | |  | | **MET2** | | | | |  | | |  | | | | |  | | | **MET3** | | | | |  | | |  | | | | |  | | |  |
| --- | --- | --- | --- | --- | --- | --- | --- | --- | --- | --- | --- | --- | --- | --- | --- | --- | --- | --- | --- | --- | --- | --- | --- | --- | --- | --- | --- | --- | --- | --- | --- | --- | --- | --- | --- | --- | --- | --- | --- | --- | --- | --- | --- | --- |
|  |  | **0 hpi** | | | **4 hpi** | | **24 hpi** | | | **48 hpi** | | **0 hpi** | | | | | **4 hpi** | | | **24 hpi** | | | | | **48 hpi** | | | **0 hpi** | | | | | **4 hpi** | | | **24 hpi** | | | | | **48 hpi** | | |  |
| **Hematocrit** | **(%)** | 30.38 ± 9.62 | | | | 23.40 ± 6.19 | | 23.75 ± 3.37 | | | 25.60 ± 8.08 | | 23.56 ± 7.07 | | | | | 23.33 ± 5.17 | | | 25.00 ± 5.21 | | | | | 23.50 ± 3.15 | | | 26.44 ± 6.25 | | | | | 27.00 ± 4.75 | | | 23.44 ± 2.60 | | | | | 25.50 ± 6.52 | |  |
| **Hemoglobin** | **(g dl^-1^)** | 0.94 ± 0.25 | | | | 0.86 ± 0.31 | | 0.93 ± 0.20 | | | 0.94 ± 0.24 | | 0.87 ± 0.30 | | | | | 0.96 ± 0.18 | | | 1.01 ± 0.36 | | | | | 0.72 ± 0.30 | | | 0.59 ± 0.28 | | | | | 1.09 ± 0.22 | | | 0.92 ± 0.28 | | | | | 1.13 ± 0.34 | |  |
| **MCV** | **(µm^3^)** | 183.57 ± 58.60 | | | | 116.75 ± 4.93 | | 144.51 ± 37.77 | | | 104.51 ± 41.86 | | 170.58 ± 67.89 | | | | | 107.60 ± 12.10 | | | 129.18 ± 23.18 | | | | | 129.35 ± 34.29 | | | 215.54 ± 78.65 | | | | | 108.10 ± 12.88 | | | 124.24 ± 21.12 | | | | | 128.86 ± 50.59 | |  |
| **MCH** | **(pg cell^-1^)** | 5.92 ± 2.65 | | | | 4.20 ± 1.35 | | 5.38 ± 1.40 | | | 3.81 ± 0.81 | | 5.52 ± 1.30 | | | | | 4.69 ± 0.69 | | | 4.85 ± 0.81 | | | | | 3.44 ± 0.86 | | | 5.79 ± 2.37 | | | | | 4.14 ± 0.53 | | | 4.86 ± 1.65 | | | | | 6.09 ± 1.86 | |  |
| **MCHC** | **(g 100 ml^-1^)** | 3.37 ± 1.47 | | | | 4.46 ± 0.97 | | 4.08 ± 1.23 | | | 3.46 ± 0.56 | | 2.99 ± 0.58 | | | | | 4.20 ± 0.65 | | | 3.97 ± 0.92 | | | | | 2.79 ± 1.01 | | | 2.19 ± 0.90 | | | | | 4.32 ± 1.32 | | | 3.97 ± 1.27 | | | | | 4.80 ± 1.32 | |  |
| **RBC** | **(x10^6^ µl^-1^)** | 1.75 ± 0.56 | | | | 2.02 ± 0.45 | | 1.78 ± 0.37 | | | 2.48 ± 0.42 | | 1.64 ± 0.56 | | | | | 2.17 ± 0.41 | | | 2.07 ± 0.60 | | | | | 2.07 ± 0.61 | | | 1.31 ± 0.63 | | | | | 2.45 ± 0.30 | | | 1.93 ± 0.37 | | | | | 1.80 ± 0.69 | |  |
| **WBC** | **(x10^4^ µl^-1^)** | 5.10 ± 0.81 | | | | 6.20 ± 2.00 | | 5.14 ± 1.10 | | | 5.79 ± 1.70 | | 5.53 ± 1.46 | | | | | 6.46 ± 1.66 | | | 7.07 ± 2.02 | | | | | 4.84 ± 1.59 | | | 5.64 ± 0.93 | | | | | 7.13 ± 1.52 | | | 7.08 ± 2.44 | | | | | 5.94 ± 2.24 | |  |
| **Parameters** | | | **Diet** | **Time** | | | | | **Diet ×**  **Time** | | | | | **Time** | | | | | | | | **Diet × Time** | | | | | | | | | | | | | | | | | | | | | | |
|  |  |  |  |  |  |  |  |  |  |  |  |  |  |  |  |  |  |  |  |  |  | **CTRL** | | | | | | | | **MET2** | | | | | | | | **MET3** | | | | | | |
|  |  |  |  |  |  |  |  |  |  |  |  |  |  | **0 hpi** | **4 hpi** | **24 hpi** | | | **48 hpi** | | | **0 hpi** | **4 hpi** | **24 hpi** | | | **48 hpi** | | | **0 hpi** | **4 hpi** | **24 hpi** | | | **48 hpi** | | | **0 hpi** | **4 hpi** | **24 hpi** | | | **48 hpi** | |
| **Hematocrit** | | | ns | ns | | | | | ns | | | | | - | - | - | | | - | | | - | - | - | | | - | | | - | - | - | | | - | | | - | - | - | | | - | |
| **Hemoglobin** | | | ns | ns | | | | | 0.003 | | | | | - | - | - | | | - | | | A | - | - | | | AB | | | AB | - | - | | | B | | | Bb | a | ab | | | Aa | |
| **MCV** | | | ns | < 0.001 | | | | | ns | | | | | a | b | b | | | b | | | - | - | - | | | - | | | - | - | - | | | - | | | - | - | - | | | - | |
| **MCH** | | | ns | 0.030 | | | | | 0.030 | | | | | - | - | - | | | - | | | a | ab | a | | | Bb | | | a | a | a | | | Bb | | | - | - | - | | | A | |
| **MCHC** | | | ns | < 0.001 | | | | | 0.024 | | | | | - | - | - | | | - | | | - | - | - | | | AB | | | b | a | ab | | | Bb | | | b | a | a | | | Aa | |
| **RBC** | | | ns | < 0.001 | | | | | 0.033 | | | | | - | - |  | | | - | | | b | ab | b | | | a | | | - | - | - | | | - | | | b | a | ab | | | ab | |
| **WBC** | | | ns | 0.028 | | | | | ns | | | | | b | a | ab | | | b | | | - | - | - | | | - | | | - | - | - | | | - | | | - | - | - | | | - | |

**Table S8.** Absolute values of peripheral blood leucocytes (thrombocytes, lymphocytes, monocytes and neutrophils) of European seabass fed dietary treatments for 4 weeks (0 hpi), at 4, 24 and 48 hours post-infection. Values are presented as mean ± SD (n=9 per treatment). Different lowercase letters indicate differences among times (Two-way ANOVA; *p* ≤ 0.05).

| **Parameters** | | **CTRL** | |  | | |  | |  | | | | **MET2** | | | |  | | |  | |  | | **MET3** | |  | |  | |  | |
| --- | --- | --- | --- | --- | --- | --- | --- | --- | --- | --- | --- | --- | --- | --- | --- | --- | --- | --- | --- | --- | --- | --- | --- | --- | --- | --- | --- | --- | --- | --- | --- |
|  |  | **0 hpi** | | **4 hpi** | | | **24 hpi** | | **48 hpi** | | | | **0 hpi** | | | | **4 hpi** | | | **24 hpi** | | **48 hpi** | | **0 hpi** | | **4 hpi** | | **24 hpi** | | **48 hpi** | |
| **Thrombocytes** | **(×10^4^ µl^-1^)** | 4.13 ± 0.56 | | | 5.25 ± 1.82 | | | 4.11 ± 1.05 | | 4.37 ± 0.93 | | | | 4.09 ± 1.15 | | | | 5.54 ± 1.68 | | | 5.18 ± 1.11 | | 4.50 ± 1.05 | | 3.82 ± 0.81 | | 5.10 ± 1.13 | | 5.23 ± 1.21 | | 4.49 ± 1.81 |
| **Lymphocytes** |  | 1.10 ± 0.16 | | | 0.68 ± 0.39 | | | 0.40 ± 0.71 | | 0.80 ± 0.96 | | | | 1.38 ± 0.86 | | | | 0.72 ± 0.39 | | | 1.16 ± 1.22 | | 0.52 ± 0.06 | | 1.51 ± 0.29 | | 1.31 ± 0.63 | | 0.92 ± 1.01 | | 0.96 ± 0.95 |
| **Monocytes** |  | 0.06 ± 0.04 | | | 0.15 ± 0.07 | | | 0.30 ± 0.21 | | 0.44 ± 0.43 | | | | 0.06 ± 0.05 | | | | 0.16 ± 0.12 | | | 0.25 ± 0.20 | | 0.56 ± 0.21 | | 0.12 ± 0.05 | | 0.13 ± 0.10 | | 0.29 ± 0.18 | | 0.31 ± 0.16 |
| **Neutrophils** |  | 0.08 ± 0.05 | | | 0.06 ± 0.05 | | | 0.13 ± 0.07 | | 0.16 ± 0.12 | | | | 0.08 ± 0.04 | | | | 0.08 ± 0.08 | | | 0.24 ± 0.14 | | 0.23 ± 0.08 | | 0.10 ± 0.06 | | 0.14 ± 0.10 | | 0.32 ± 0.25 | | 0.12 ± 0.12 |
| **Parameters** | | **Diet** | **Time** | | | **Diet ×**  **Time** | | | | | **Time** | | | | | | | |  |  |  |  |  |  |  |  |  |  |  |  |  |
|  |  |  |  |  |  |  |  |  |  |  |  |  |  |  |  |  |  |  |  |  |  |  |  |  |  |  |  |  |  |  |  |
|  |  |  |  |  |  |  |  |  |  |  | **0 hpi** | **4 hpi** | | | **24 hpi** | **48 hpi** | | |  |  |  |  |  |  |  |  |  |  |  |  |  |
| **Thrombocytes** | | ns | 0.010 | | | ns | | | | | b | a | | | ab | ab | | |  |  |  |  |  |  |  |  |  |  |  |  |  |
| **Lymphocytes** | | ns | ns | | | ns | | | | | - | - | | | - | - | | |  |  |  |  |  |  |  |  |  |  |  |  |  |
| **Monocytes** | | ns | < 0.001 | | | ns | | | | | c | bc | | | ab | a | | |  |  |  |  |  |  |  |  |  |  |  |  |  |
| **Neutrophils** | | ns | < 0.001 | | | ns | | | | | b | b | | | a | ab | | |  |  |  |  |  |  |  |  |  |  |  |  |  |

**Table S9.** Skin mucus immune parameters (peroxidase, protease and lysozyme activities, and IgM levels) of European seabass fed dietary treatments for 4 weeks (0 hpi), at 4, 24 and 48 hours post-infection. Values are presented as mean ± SD (n=3 per treatment). Different lowercase letters indicate differences among times (Two-way ANOVA; *p* ≤ 0.05).

| **Parameters** | | | **CTRL** | |  | |  | |  | | | | **MET2** | | | |  | | |  | |  | | **MET3** | |  | |  | |  | |
| --- | --- | --- | --- | --- | --- | --- | --- | --- | --- | --- | --- | --- | --- | --- | --- | --- | --- | --- | --- | --- | --- | --- | --- | --- | --- | --- | --- | --- | --- | --- | --- |
|  |  |  | **0 hpi** | | **4 hpi** | | **24 hpi** | | **48 hpi** | | | | **0 hpi** | | | | **4 hpi** | | | **24 hpi** | | **48 hpi** | | **0 hpi** | | **4 hpi** | | **24 hpi** | | **48 hpi** | |
| **Peroxidase** | **(units mg^-1^ protein)** | | 26.36 ± 10.95 | | 31.45 ± 4.77 | | | 19.39 ± 6.27 | | 39.01 ± 7.93 | | | | 23.84 ± 5.46 | | | | 31.50 ± 8.85 | | | 42.36 ± 10.00 | | 57.21 ± 28.38 | | 23.57 ± 9.09 | | 34.09 ± 7.10 | | 27.65 ± 5.64 | | 48.42 ± 29.02 |
| **Protease** | **(%)** | | 1.39 ± 0.10 | | 1.73 ± 0.13 | | | 15.13 ± 4.24 | | 7.30 ± 3.75 | | | | 1.17 ± 0.28 | | | | 2.39 ± 1.07 | | | 7.76 ± 5.41 | | 10.30 ± 3.08 | | 0.96 ± 0.23 | | 2.46 ± 0.52 | | 12.85 ± 7.43 | | 8.74 ± 1.49 |
| **Lysozyme** | **(µg mg^-1^ protein)** | | 6.02 ± 4.86 | | 7.05 ± 1.02 | | | 1.94 ± 0.91 | | 4.80 ± 0.91 | | | | 3.62 ± 1.66 | | | | 5.67 ± 1.60 | | | 3.96 ± 1.56 | | 8.47 ± 6.51 | | 6.80 ± 3.91 | | 3.65 ± 0.64 | | 2.32 ± 1.21 | | 7.54 ± 5.09 |
| **IgM** | **(OD)** | | 0.02 ± 0.02 | | 0.05 ± 0.04 | | | 0.08 ± 0.04 | | 0.09 ± 0.02 | | | | 0.03 ± 0.02 | | | | 0.03 ± 0.04 | | | 0.09 ± 0.03 | | 0.17 ± 0.10 | | 0.03 ± 0.01 | | 0.05 ± 0.03 | | 0.09 ± 0.04 | | 0.13 ± 0.05 |
| **Parameters** | | **Diet** | | **Time** | | **Diet ×**  **Time** | | | | | **Time** | | | | | | | |  |  |  |  |  |  |  |  |  |  |  |  |  |
|  |  |  |  |  |  |  |  |  |  |  |  |  |  |  |  |  |  |  |  |  |  |  |  |  |  |  |  |  |  |  |  |
|  |  |  |  |  |  |  |  |  |  |  | **0 hpi** | **4 hpi** | | | **24 hpi** | **48 hpi** | | |  |  |  |  |  |  |  |  |  |  |  |  |  |
| **Peroxidase** | | ns | | 0.005 | | ns | | | | | b | ab | | | b | a | | |  |  |  |  |  |  |  |  |  |  |  |  |  |
| **Protease** | | ns | | < 0.001 | | ns | | | | | c | b | | | a | a | | |  |  |  |  |  |  |  |  |  |  |  |  |  |
| **Lysozyme** | | ns | | 0.005 | | ns | | | | | ab | a | | | b | a | | |  |  |  |  |  |  |  |  |  |  |  |  |  |
| **IgM** | | ns | | < 0.001 | | ns | | | | | c | bc | | | ab | a | | |  |  |  |  |  |  |  |  |  |  |  |  |  |

**Table S10.** Intestine oxidative stress biomarkers, catalase activity (CAT) and lipid peroxidation levels (LPO), of European seabass fed dietary treatments for 4 weeks (0 hpi), at 4, 24 and 48 hours post-infection. Values are presented as mean ± SD (n=6 per treatment). Different lowercase letters indicate differences among times (Two-way ANOVA; *p* ≤ 0.05).

| **Parameters** | | | **CTRL** | |  | |  | |  | | | | **MET2** | | | |  | | |  | |  | | **MET3** | |  | |  | |  | |
| --- | --- | --- | --- | --- | --- | --- | --- | --- | --- | --- | --- | --- | --- | --- | --- | --- | --- | --- | --- | --- | --- | --- | --- | --- | --- | --- | --- | --- | --- | --- | --- |
|  |  |  | **0 hpi** | | **4 hpi** | | **24 hpi** | | **48 hpi** | | | | **0 hpi** | | | | **4 hpi** | | | **24 hpi** | | **48 hpi** | | **0 hpi** | | **4 hpi** | | **24 hpi** | | **48 hpi** | |
| **CAT** | **(units mg^-1^ protein)** | | 57.21 ± 11.89 | | 58.45 ± 12.28 | | | 46.63 ± 2.68 | | 41.59 ± 3.11 | | | | 56.08 ± 3.70 | | | | 52.54 ± 11.21 | | | 54.29 ± 21.41 | | 37.07 ± 10.40 | | 61.08 ± 9.91 | | 56.15 ± 8.00 | | 49.33 ± 9.29 | | 47.34 ± 5.14 |
| **LPO** | **(nmol g^-1^ wt)** | | 89.35 ± 13.15 | | 113.62 ± 35.32 | | | 82.20 ± 31.70 | | 100.29 ± 21.42 | | | | 78.28 ± 14.22 | | | | 107.76 ± 44.84 | | | 67.60 ± 16.26 | | 91.05 ± 19.87 | | 77.65 ± 17.11 | | 74.22 ± 17.38 | | 96.16 ± 35.53 | | 76.57 ± 11.78 |
| **Parameters** | | **Diet** | | **Time** | | **Diet ×**  **Time** | | | | | **Time** | | | | | | | |  |  |  |  |  |  |  |  |  |  |  |  |  |
|  |  |  |  |  |  |  |  |  |  |  |  |  |  |  |  |  |  |  |  |  |  |  |  |  |  |  |  |  |  |  |  |
|  |  |  |  |  |  |  |  |  |  |  | 0 hpi | 4 hpi | | | 24 hpi | 48 hpi | | |  |  |  |  |  |  |  |  |  |  |  |  |  |
| **CAT** | | ns | | < 0.001 | | ns | | | | | a | a | | | ab | b | | |  |  |  |  |  |  |  |  |  |  |  |  |  |
| **LPO** | | ns | | ns | | ns | | | | | - | - | | | - | - | | |  |  |  |  |  |  |  |  |  |  |  |  |  |

**Table S11.** Quantitative expression of *cd209c*, *cd8b*, *il1b*, *il6*, *il8*, *mmp9*, *nfkb*, *sms*, *tgfb* and *tnfa* in the head kidney of European seabass fed dietary treatments for 4 weeks (0 hpi), at 4, 24 and 48 hours post-infection. Values are presented as mean ± SD (n=6 per treatment). Different capital letters stand for differences among dietary treatments and different lowercase letters indicate differences among times (Two-way ANOVA; *p* ≤ 0.05).

| **Genes** | **CTRL** | | |  | |  | |  | | | **MET2** | |  | | |  | | | |  | | | **MET3** | | |  | |  | | | |  | | | |
| --- | --- | --- | --- | --- | --- | --- | --- | --- | --- | --- | --- | --- | --- | --- | --- | --- | --- | --- | --- | --- | --- | --- | --- | --- | --- | --- | --- | --- | --- | --- | --- | --- | --- | --- | --- |
|  | **0 hpi** | | | **4 hpi** | | **24 hpi** | | **48 hpi** | | | **0 hpi** | | **4 hpi** | | | **24 hpi** | | | | **48 hpi** | | | **0 hpi** | | | **4 hpi** | | | **24 hpi** | | | | **48 hpi** | | |
| ***cd209c*** | 0.77 ± 0.35 | | | 22.28 ± 12.15 | | 2718.47 ± 1083.60 | | 3177.94 ± 2407.01 | | | 0.45 ± 0.15 | | 39.32 ± 26.14 | | | 1748.92 ± 2047.95 | | | | 3805.84 ± 3098.37 | | | 2.09 ± 1.80 | | | 22.34 ± 22.75 | | | 1164.95 ± 1089.01 | | | | 1309.76 ± 1960.60 | | |
| ***cd8b*** | 1.32 ± 0.97 | | | 0.81 ± 0.37 | | 0.70 ± 0.38 | | 0.56 ± 0.22 | | | 2.43 ± 1.37 | | 1.83 ± 1.69 | | | 0.39 ± 0.15 | | | | 0.53 ± 3.13 | | | 0.80 ± 0.31 | | | 0.37 ± 0.10 | | | 0.86 ± 0.50 | | | | 0.43 ± 0.28 | | |
| ***il1b*** | 1.40 ± 1.19 | | | 29.88 ± 36.45 | | 60.14 ± 45.69 | | 30.07 ± 14.56 | | | 1.25 ± 0.76 | | 7.40 ± 7.52 | | | 47.40 ± 40.78 | | | | 46.66 ± 29.91 | | | 2.14 ± 1.75 | | | 0.95 ± 0.44 | | | 27.02 ± 16.26 | | | | 26.83 ± 28.21 | | |
| ***il6*** | 0.85 ± 0.18 | | | 1.91 ± 0.82 | | 8.23 ± 4.16 | | 8.09 ± 5.20 | | | 0.91 ± 0.37 | | 1.50 ± 0.26 | | | 6.23 ± 1.56 | | | | 8.63 ± 3.40 | | | 0.78 ± 0.24 | | | 1.12 ± 0.42 | | | 6.09 ± 2.15 | | | | 5.22 ± 3.29 | | |
| ***il8*** | 1.35 ± 1.15 | | | 7.65 ± 8.03 | | 56.40 ± 43.52 | | 37.17 ± 19.23 | | | 1.17 ± 0.69 | | 6.15 ± 5.59 | | | 28.51 ± 18.83 | | | | 73.30 ± 25.08 | | | 0.92 ± 0.46 | | | 2.15 ± 0.90 | | | 19.25 ± 10.58 | | | | 30.47 ± 28.22 | | |
| ***mmp9*** | 1.21 ± 0.82 | | | 3.43 ± 1.05 | | 2.60 ± 1.09 | | 5.38 ± 2.26 | | | 0.72 ± 0.29 | | 2.24 ± 0.59 | | | 4.02 ± 2.06 | | | | 4.42 ± 2.26 | | | 1.16 ± 0.75 | | | 1.11 ± 0.31 | | | 2.55 ± 0.94 | | | | 4.45 ± 1.80 | | |
| ***nfkb*** | 1.02 ± 0.22 | | | 1.13 ± 0.41 | | 4.91 ± 1.77 | | 7.45 ± 3.89 | | | 0.72 ± 0.21 | | 1.12 ± 0.59 | | | 4.19 ± 1.61 | | | | 6.29 ± 1.57 | | | 0.66 ± 0.32 | | | 0.59 ± 0.24 | | | 4.67 ± 2.47 | | | | 5.39 ± 2.66 | | |
| ***sms*** | 1.13 ± 0.65 | | | 1.12 ± 0.18 | | 2.12 ± 0.95 | | 3.41 ± 1.19 | | | 0.76 ± 0.26 | | 1.24 ± 0.47 | | | 1.92 ± 0.77 | | | | 3.41 ± 0.79 | | | 0.59 ± 0.08 | | | 0.97 ± 0.32 | | | 1.85 ± 0.67 | | | | 3.37 ± 0.81 | | |
| ***tgfb*** | 1.05 ± 0.36 | | | 0.82 ± 0.13 | | 1.52 ± 0.44 | | 2.94 ± 0.37 | | | 0.84 ± 0.22 | | 1.04 ± 0.24 | | | 1.19 ± 0.10 | | | | 2.85 ± 0.58 | | | 0.78 ± 0.47 | | | 0.70 ± 0.07 | | | 1.54 ± 0.29 | | | | 2.54 ± 0.43 | | |
| ***tnfa*** | 0.71 ± 0.32 | | | 1.91 ± 0.72 | | 2.49 ± 0.85 | | 3.92 ± 1.05 | | | 0.99 ± 0.58 | | 1.64 ± 0.58 | | | 7.17 ± 4.80 | | | | 4.35 ± 1.14 | | | 0.75 ± 0.14 | | | 0.93 ± 0.34 | | | 3.51 ± 2.12 | | | | 5.63 ± 3.26 | | |
| **Genes** | | **Diet** | **Time** | | **Diet ×**  **Time** | | **Diet** | | | | | **Time** | | | | | | **Diet × Time** | | | | | | | | | | | | | | | | | |
|  |  |  |  |  |  |  |  |  |  |  |  |  |  |  |  |  |  | **CTRL** | | | | | | **MET2** | | | | | | **MET3** | | | | | |
|  |  |  |  |  |  |  | **CTRL** | | **MET2** | **MET3** | | **0 hpi** | | **4 hpi** | **24 hpi** | | **48 hpi** | **0 hpi** | **4 hpi** | | **24 hpi** | **48 hpi** | | **0 hpi** | **4 hpi** | | **24 hpi** | **48 hpi** | | **0 hpi** | **4 hpi** | | | **24 hpi** | **48 hpi** |
| ***cd209c*** | | ns | < 0.001 | | ns | | - | | - | - | | c | | b | a | | a | - | - | | - | - | | - | - | | - | - | | - | - | | | - | - |
| ***cd8b*** | | 0.038 | < 0.001 | | 0.012 | | - | | - | - | | - | | - | - | | - | - | AB | | - | - | | a | Aab | | c | bc | | - | B | | | - | - |
| ***il1b*** | | 0.020 | < 0.001 | | ns | | A | | AB | B | | c | | b | a | | a | - | - | | - | - | | - | - | | - | - | | - | - | | | - | - |
| ***il6*** | | 0.013 | < 0.001 | | ns | | A | | AB | B | | c | | b | a | | a | - | - | | - | - | | - | - | | - | - | | - | - | | | - | - |
| ***il8*** | | 0.016 | < 0.001 | | ns | | A | | AB | B | | c | | b | a | | a | - | - | | - | - | | - | - | | - | - | | - | - | | | - | - |
| ***mmp9*** | | ns | < 0.001 | | 0.024 | | - | | - | - | | - | | - | - | | - | b | Aa | | a | a | | b | Aa | | a | a | | c | Bbc | | | ab | a |
| ***nfkb*** | | 0.010 | < 0.001 | | ns | | A | | AB | B | | c | | b | a | | a | - | - | | - | - | | - | - | | - | - | | - | - | | | - | - |
| ***sms*** | | ns | < 0.001 | | ns | | - | | - | - | | d | | c | b | | a | - | - | | - | - | | - | - | | - | - | | - | - | | | - | - |
| ***tgfb*** | | ns | < 0.001 | | ns | | - | | - | - | | c | | c | b | | a | - | - | | - | - | | - | - | | - | - | | - | - | | | - | - |
| ***tnfa*** | | 0.028 | < 0.001 | | 0.010 | | - | | - | - | | - | | - | - | | - | c | Ab | | Bab | a | | b | ABb | | Aa | a | | b | Bb | | | ABa | a |
